# Unveiling the therapeutic potential of natural products in Alzheimer’s disease: insights from *in vitro*, *in vivo*, and clinical studies

**DOI:** 10.3389/fphar.2025.1601712

**Published:** 2025-06-23

**Authors:** Nahida Aktary, Yerim Jeong, Seungji Oh, Yeju Shin, Yoonsoo Sung, Muntajin Rahman, Livia Ramos Santiago, Jinwon Choi, Han Gyeul Song, Fahrul Nurkolis, Rosy Iara Maciel Azambuja Ribeiro, Moon Nyeo Park, Bonglee Kim

**Affiliations:** ^1^ College of Korean Medicine, Kyung Hee University, Seoul, Republic of Korea; ^2^ Department of Biological Sciences, State Islamic University of Sunan Kalijaga (UIN Sunan Kalijaga), Yogyakarta, Indonesia; ^3^ Experimental Pathology Laboratory, Midwest Campus, Federal University of São João del-Rei, Divinópolis, Brazil

**Keywords:** Alzheimer’s disease (AD), natural products, network pharmacology, nutritional deficiencies, neuroprotective, neurodegenerative

## Abstract

Alzheimer’s disease (AD) is a multifactorial neurodegenerative disorder described as progressive cognitive decline and neuronal dysfunction, affecting millions globally. While current pharmacological treatments provide symptomatic relief and modestly slow disease progression, they fail to address the underlying pathophysiology and are often accompanied by severe adverse effects. This underscores the urgent need for innovative, multi-target therapeutic strategies that can effectively step in AD’s complex pathogenesis. Emerging evidence highlights the therapeutic potential of natural products, particularly herbal medicines, as versatile modulators of key pathogenic processes in AD. These compounds exert neuroprotective effects by mitigating oxidative stress, suppressing neuroinflammation, inhibiting tau hyperphosphorylation, and reducing amyloid-beta aggregation. Additionally, they strengthen synaptic plasticity and stabilize mitochondrial function, offering a holistic approach to disease control. This comprehensive review synthesizes findings from network pharmacology, *in vitro* and *in vivo* studies, and clinical trials to evaluate the role of natural products in AD treatment. Advances in bioinformatics and systems biology facilitate the mapping of intricate protein-protein interactions, the identification of potential biomarkers, and the clarification of molecular mechanisms underlying AD progression. Integrating phytochemicals with conventional AD medications may improve therapeutic efficacy through synergistic mechanisms; however, pharmacokinetic interactions and safety considerations must be rigorously assessed. Notably, clinical trials investigating compounds such as curcumin, resveratrol, and ginsenosides suggest promising adjunctive benefits when incorporated into established treatment regimens. Furthermore, the convergence of herbal therapeutics with modern pharmacology presents an avenue for customized and integrative AD management. This review also emphasizes advancements in experimental models, including brain organoids and transgenic animals, which serve as crucial platforms for mechanistic studies and therapeutic validation. Ongoing trials on plant-derived compounds continue to pave the way for translational applications, reinforcing the viability of natural product-based interventions. By advocating a multidisciplinary framework that merges traditional medicine, modern pharmacology, and precision medicine, this work contributes to reshaping the AD landscape of therapy. It provides a roadmap for future research, fostering novel treatment paradigms that prioritize efficacy, safety, and sustainability in combating this disastrous disorder.

## 1 Introduction

Alzheimer’s disease (AD) is a progressive and multifactorial neurodegenerative disorder that predominantly affects the elderly population, manifesting as severe cognitive decline, memory impairment, and behavioral disturbance (Tripathi et al., 2024). Pathologically, AD is defined by the extracellular accumulation of amyloid beta (Aβ) plaques and the intracellular formation of neurofibrillary tangles composed of hyperphosphorylated tau proteins. These hallmark abnormalities disrupt synaptic communication, trigger neuroinflammation, and promote neuronal apoptosis, collectively leading to extensive neurodegeneration and cognitive dysfunction. Despite decades of intensive research, the exact mechanisms driving the onset and progression of AD remain incompletely understood, underscoring the urgent need for continued investigation into its pathophysiology and the development of effective therapeutic interventions (Korczyn and Grinberg, 2024). As the global population ages, the prevalence of AD is rapidly increasing, imposing a significant burden on patients, caregivers, and healthcare systems. This growing public health challenge highlights the limitations of existing therapies and the pressing demand for disease-modifying treatments that go beyond symptomatic relief (Wang et al., 2024). According to the Alzheimer’s Association, dementia—including AD—is the seventh leading cause of death worldwide, underscoring its profound societal and economic impact (Collaborators et al., 2021). The complexity of AD pathogenesis lies in its convergence of interdependent pathological processes, including oxidative stress, chronic neuroinflammation, mitochondrial dysfunction, and impaired autophagy (Park et al., 2022). These mechanisms are not isolated but rather amplify one another, necessitating a comprehensive therapeutic approach capable of targeting multiple pathological pathways simultaneously. In this context, natural compounds have emerged as promising candidates for AD treatment, owing to their capacity to modulate several interconnected mechanisms. Oxidative stress, driven by excessive production of reactive oxygen species (ROS), leads to damage of neuronal lipids, proteins, and DNA (Guo et al., 2020). Chronic neuroinflammation—mediated by activated microglia and astrocytes—exacerbates neuronal injury through the release of pro-inflammatory cytokines such as TNF-α and IL-1β (Singh et al., 2019). Mitochondrial dysfunction, a key feature of AD, further exacerbates ROS production, impairs ATP generation, disrupts calcium homeostasis, and ultimately induces neuronal apoptosis (Bhatt et al., 2021). Current pharmacological therapies, including cholinesterase inhibitors (ChEIs) (e.g., donepezil, rivastigmine, galantamine) and N-methyl-D-aspartate (NMDA) receptor antagonists (e.g., memantine), offer only modest symptomatic relief without altering disease progression (Butterfield and Halliwell, 2019). Moreover, their limited tolerability, adverse effects, and variable patient responses emphasize the urgent need for safer, more effective, and disease-modifying therapeutic strategies (Zhang et al., 2024).

Recent advancements in neuropharmacology have intensified the search for multi-targeted therapeutic agents, particularly plant-derived compounds that exhibit neuroprotective, anti-inflammatory, and antioxidant properties (Papadopoulou et al., 2024). These natural products have garnered increasing attention due to their ability to modulate multiple pathological features of AD, including oxidative stress, mitochondrial dysfunction, amyloid-beta aggregation, and neuroinflammation. The integration of systems biology and pharmacogenomic tools has further enabled researchers to incorporate these agents into personalized medicine frameworks, tailoring interventions to individual molecular and genetic profiles (Tohda et al., 2017). While numerous reviews have investigated the effects of natural compounds in AD, they often focus narrowly on specific biochemical pathways or compound classes, lacking a unified translational perspective (Zhang X. et al., 2019). In contrast, recent studies underscore the value of neurofilaments—key structural proteins critical for axonal stability and intracellular transport—as emerging biomarkers in neurodegenerative disorders such as Alzheimer’s, Parkinson’s, and Huntington’s disease. Their elevated presence in cerebrospinal fluid and peripheral blood following neuroaxonal injury offers important diagnostic and prognostic utility (Sharma et al., 2024). This review distinguishes itself by providing a comprehensive synthesis of evidence from *in vitro*, *in vivo*, and clinical investigations, while also incorporating cutting-edge advances in brain organoid modeling and network pharmacology. Moreover, it evaluates the potential of combinatorial natural product therapies within precision medicine frameworks, aiming to address the multifactorial complexity of AD. By embracing a multidisciplinary approach that merges traditional knowledge with modern pharmacological and systems-level tools, this work contributes to the evolving landscape of AD therapeutics and supports the integration of natural compounds into next-generation, individualized treatment strategies (Yusof and Fauzi, 2024; Zhao et al., 2024).

### 1.1 Emerging role of dietary interventions

Recent research suggests that certain dietary patterns can influence essential pathophysiological characteristics of AD, such as neuroinflammation, oxidative stress, and amyloid-beta deposition (Stefaniak et al., 2022). Nutritional methods can help improve brain resilience by increasing synaptic plasticity, modulating insulin signaling, and lowering the burden of neurotoxic proteins.

#### 1.1.1 Ketogenic diet (KD)

The standard ketogenic diet (KD), originally devised to treat epilepsy, is high-fat, low-carbohydrate, typically with a 4:1 or 3:1 ratio of fats to carbohydrates and protein. The KD causes a state of physiological ketosis, altering brain energy metabolism from glucose to ketone bodies like β-hydroxybutyrate and acetoacetate (Crosby et al., 2021). This metabolic shift is neuroprotective, reducing oxidative stress, inhibiting Aβ plaque formation, attenuating neuroinflammation, and improving cognitive performance in animal models and early clinical investigations (Rusek et al., 2019; Lilamand et al., 2020; Ali et al., 2024; Kwon et al., 2024). Preclinical and human evidence indicate that KD may improve mitochondrial function, calm mood, and delay neurodegeneration (Jain and Vohora, 2025).

#### 1.1.2 Mediterranean diet (MD)

The Mediterranean Diet (MD) stresses the high intake of plant-based foods such as vegetables, fruits, legumes, whole grains, and nuts, with olive oil serving as the main source of dietary fat (Scarmeas et al., 2006; Calil et al., 2018; Vassilaki et al., 2018; Karstens et al., 2019). It also entails moderate consumption of fish and dairy, limited intake of red and processed meats, and optional moderate wine consumption with meals (Scarmeas et al., 2009). The MD was initially recognized for cardiovascular protection, but it has also been shown to boost cognitive health and reduce the risk of AD. Adherence to the MD has been linked to shorter cognitive decline, enhanced memory, and a reduced incidence of moderate cognitive impairment and dementia. A meta-analysis of prospective cohort studies demonstrated that strong adherence to the MD substantially lowers the risk of cognitive impairment and AD in older persons (Coelho-Júnior et al., 2021). The neuroprotective effects of the MD are likely due to synergistic actions of antioxidants, anti-inflammatory drugs, and vascular modulators included in the diet (García-Casares et al., 2021). This diet increases systemic anti-inflammatory and antioxidant effects, decreases insulin resistance, and improves cerebrovascular health all relevant in AD etiology (Cremonini et al., 2019).

#### 1.1.3 MIND diet

Morris et colleagues. created the MIND diet, which stands for Mediterranean-DASH Intervention for Neurodegenerative Delay, to particularly address age-related cognitive decline (Morris et al., 2015). It combines the Mediterranean and DASH (Dietary Approaches to Stop Hypertension) diets, emphasizing brain-protective foods such as leafy greens, berries, whole grains, olive oil, almonds, and fish, while reducing red meats, butter, pastries, and fried meals (Levak et al., 2024). Numerous cohort studies have confirmed that higher adherence to the MIND diet is associated with slower cognitive aging, reduced incidence of AD, and even protection against Parkinsonian syndromes (Włodarek, 2019). Recent neuroimaging studies suggest that adherence to the MIND diet correlates with preserved gray matter volume and cortical thickness, although more longitudinal evidence is warranted (Staubo et al., 2017). Future research that combines APOE genotyping with dietary responsiveness might open the door for tailored nutritional therapy in at risk groups. The MIND diet has been proven to lessen the risk of AD by up to 53% in highly adherent people and by 35% with moderate adherence (Vu et al., 2022).

#### 1.1.4 Plant based diets

**FIGURE 1:**
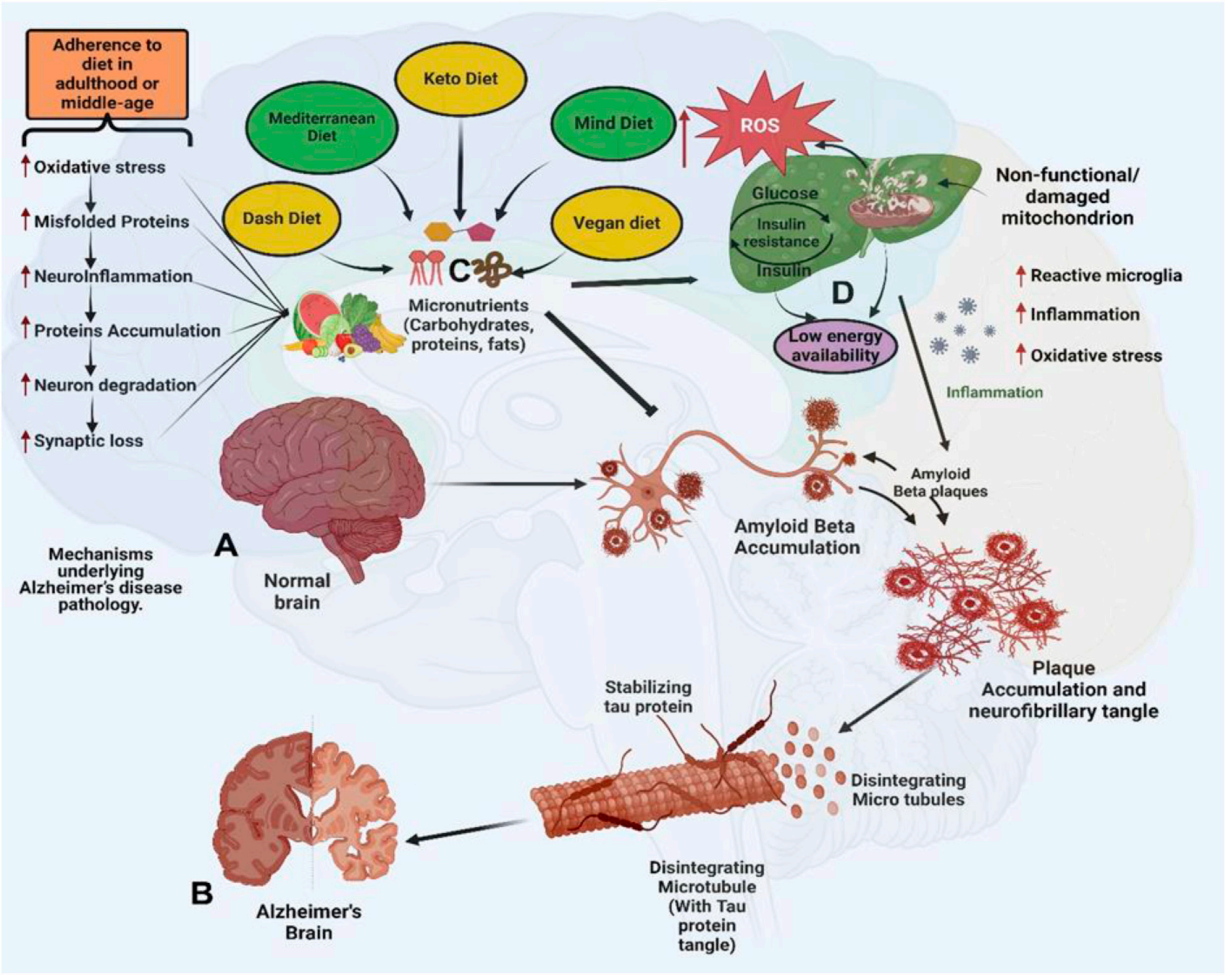
Anatomical and Molecular Features of Alzheimer’s Disease (AD) and the Impact of Dietary Patterns. **(A)** Illustrates a healthy brain with intact neuronal structures, showcasing normal synaptic function, mitochondrial activity, and absence of pathological protein aggregates. **(B)** Depicts an AD-affected brain, marked by the accumulation of extracellular amyloid-beta (Aβ) plaques and intracellular neurofibrillary tangles formed by hyperphosphorylated tau protein. These pathological hallmarks are accompanied by widespread neuronal loss, synaptic degradation, and brain atrophy. **(C)** Dietary patterns such as the DASH diet, ketogenic diet, Mediterranean diet, MIND diet, and vegan diet are proposed to modulate AD pathology by lowering blood pressure and oxidative stress, enhancing mitochondrial function, reducing amyloid-beta (Aβ) production, providing neuroprotective omega-3 fatty acids, and delaying cognitive decline. Epidemiological studies have shown that adherence to the Mediterranean diet is associated with a 25%–48% reduced risk of developing AD, while the DASH diet is linked to a 39% slower rate of cognitive decline. Preclinical studies in animal models have demonstrated that ketogenic diet interventions can reduce Aβ plaque burden by approximately 25%. **(D)** In the AD brain, mitochondrial dysfunction resulting from impaired insulin signaling and disrupted glucose metabolism leads to excessive production of reactive oxygen species (ROS). The accumulation of ROS triggers oxidative stress and neuroinflammation, which in turn accelerate neuronal damage and apoptosis. These interrelated processes—mitochondrial impairment, oxidative stress, and chronic inflammation—synergistically exacerbate synaptic dysfunction and cognitive decline.

Growing evidence supports the role of plant-based dietary patterns in preventing AD and Alzheimer’s disease-related dementias (ADRD), through both direct neuroprotective effects and modulation of systemic risk factors (Kheirouri et al., 2024). Plant-rich diets have consistently been related to a lower incidence of cognitive decline, decreased neuroinflammation, and slower development of AD (de Crom et al., 2023). These advantages are derived from combinatorial actions enhancing brain antioxidant capacity while concurrently lowering cardiometabolic risk factors such as insulin resistance, obesity, and hypertension (Ellouze et al., 2023). Plant-based diets offer a holistic approach to AD prevention by modulating both CNS-specific and systemic pathways. Major public health organizations, such as the American Heart Association and the United States Dietary Guidelines, aggressively advocate for plant-based eating patterns to improve cardiovascular and cognitive health. Dietary adherence has been associated with better global cognition, decreased brain atrophy, and slower cognitive aging in large population-based research (Omar 2019; Raval et al., 2020; Sharma, 2021; Ellouze et al., 2023). Tailored dietary interventions, particularly those focusing on polyphenol-rich foods, omega-3 fatty acids, and low glycemic load, are emerging as viable supplements to traditional Alzheimer’s treatments. Personalized techniques that take into account genetic predisposition (e.g., APOE4) and metabolic state may further increase the efficacy of plant-based intervention (Bolengo, 2023; Liang et al., 2023; Warren et al., 2024). However, it is important to acknowledge that much of the current evidence supporting the efficacy of dietary interventions in AD is derived from preclinical models and observational cohort studies, both of which are inherently limited in their ability to establish causal relationships. Although numerous associations between specific dietary patterns and improved cognitive outcomes have been reported, findings from randomized controlled trials (RCTs) remain inconsistent. For instance, Lilamand et al. (2020) questioned the clinical relevance of such interventions after observing no significant cognitive improvements in AD patients following adherence to a Mediterranean diet. These discrepancies highlight the urgent need for rigorously designed, long-term clinical trials with well-defined cognitive endpoints and standardized dietary protocols. Addressing these limitations is essential for validating the role of dietary strategies as potential disease-modifying approaches in AD management. Figure 1 illustrates how diet interfaces with the multifactorial pathophysiology of AD, highlighting anatomical vulnerability, molecular cascades, and potential dietary interventions.

### 1.2 Types of Alzheimer’s disease

AD has several subgroups depending on age of onset, genetic predisposition, and clinical characteristics. Understanding these categories is crucial for creating precise diagnoses and therapies.

#### 1.2.1 Early-onset Alzheimer’s disease (EOAD)

EOAD typically occurs before the age of 65 and constitutes less than 10% of all AD cases. *It often involves autosomal dominant mutations in genes such as amyloid precursor protein (APP), presenilin 1 (PSEN1), and presenilin2 (PSEN2), which accelerate amyloid-beta accumulation and early pathological changes.* Clinically, EOAD progresses rapidly and may present with atypical symptoms such as visuospatial dysfunction, apraxia, or language impairment (Marshe et al., 2019; Rezazadeh et al., 2019; Bertoux et al., 2020; Ramanan et al., 2020; De Felice et al., 2022; Valdez-Gaxiola et al., 2024).

#### 1.2.2 Late-onset Alzheimer’s disease (LOAD)

LOAD is the most prevalent form of AD, manifesting after age 65 and accounting for the vast majority of cases. Unlike EOAD, LOAD is sporadic in nature and results from a multifactorial etiology involving age, vascular health, metabolic syndrome, and gene-environment interactions (Marshe et al., 2019; Rezazadeh et al., 2019; De Felice et al., 2022). The APOE ε4 allele remains the most significant genetic risk factor, enhancing susceptibility to both amyloid pathology and vascular dysfunction (Bonomi et al., 2024). Clinically, LOAD begins with episodic memory decline and gradually advances to involve executive dysfunction (Kelly et al., 2020) language disturbances (Paolini Paoletti et al., 2021) and impaired daily living (Guo et al., 2022).

#### 1.2.3 LOAD subtypes and atypical variants

Typical Amnestic AD: The classic presentation involves prominent memory loss due to early involvement of the hippocampus and medial temporal lobe structures (Bertoux et al., 2020; Ramanan et al., 2020). Non-Amnestic AD: This variant feature early deficits in language, visuospatial ability, or executive function, often associated with parietal or frontal cortical involvement (Phillips et al., 2019). Mixed Dementia: Mixed dementia reflects overlapping AD and cerebrovascular pathology, including Aβ plaques, tau deposition, and ischemic changes (Desta, 2021), ischemic changes (Chong et al., 2019). Parkinson-AD Overlap: Parkinson AD overlap syndrome presents with coexisting tauopathy and synucleinopathy, accompanied by blood brain barrier breakdown. Recognition of this phenotype may enable development of dual-pathway targeted therapies (Zhao et al., 2021; Hoosen et al., 2024).

### 1.3 Nutrition deficiencies in Alzheimer’s disease

Nutritional deficiencies are increasingly recognized as modifiable risk factors in AD, influencing neuronal health, vascular integrity, and cognitive function. Below, we summarize key micronutrients and biological cofactors implicated in AD pathogenesis (Bianchi, 2024).

#### 1.3.1 Vitamin B12

Vitamin B12 deficiency, prevalent in older adults, is associated with cognitive decline, memory loss, and increased dementia risk. Mechanistically, B12 deficiency leads to elevated homocysteine levels and impaired methylation pathways, contributing to neuronal toxicity and cerebrovascular damage. Diagnostic workup should include not only serum B12 but also plasma methylmalonic acid (MMA) and homocysteine levels to detect subclinical deficiency. Supplementation with B12 may delay cognitive deterioration, particularly in individuals with elevated homocysteine or low baseline B12 (Hsu et al., 2016; Sahu et al., 2022).

#### 1.3.2 Vitamin D

Vitamin D plays a neuroprotective role by regulating calcium homeostasis, modulating immune function, and attenuating amyloid beta (Aβ) and tau induced neurotoxicity (Grimm et al., 2017). Interventional trials yield mixed outcomes, but some suggest that maintaining sufficient vitamin D levels may protect against age-related cognitive impairment (Rossom et al., 2012; Schietzel et al., 2019; Bischoff-Ferrari et al., 2020; Kang et al., 2021).

#### 1.3.3 DHA and EPA (Omega-3 fatty acids)

Docosahexaenoic acid (DHA) and eicosapentaenoic acid (EPA) are essential omega-3 fatty acids involved in neuronal membrane stability, synaptic plasticity, and anti-inflammatory signaling (Zhang E. et al., 2016). Lower blood levels of DHA have been linked to hippocampal atrophy, poor cognition, and increased amyloid accumulation (Yassine et al., 2016). Modern Western diets are often deficient in n-3 fatty acids and disproportionately high in n-6 fatty acids such as arachidonic acid, contributing to a pro-inflammatory state (de Magalhães et al., 2016). Supplementation with DHA/EPA may reduce amyloid burden, mitigate tau pathology, and improve memory in animal and early-phase clinical studies (Arellanes et al., 2020).

#### 1.3.4 Transitional metal ions

Dysregulation of essential trace metals particularly iron (Fe), copper (Cu), and zinc (Zn) is increasingly recognized in AD pathophysiology. Excess accumulation of Fe, Cu, and Zn in the AD brain enhances oxidative stress, facilitates Aβ aggregation, and promotes tau hyperphosphorylation (Eisenstein, 2000). These metals are often co-localized with amyloid plaques and may directly influence neurotoxicity through redox reactions (Singh et al., 2024). Moreover, the blood-brain barrier (BBB) plays a crucial role in regulating brain metal homeostasis. Disruption of metal transport proteins can lead to both toxic accumulation and essential deficiencies (Zheng and Monnot, 2012).Chelation therapies and metal modulating nutraceuticals are currently under investigation as potential therapeutic options in AD (Sensi et al., 2018). Although many dietary deficiencies are associated with the pathophysiology of AD, findings from interventional studies have been conflicting or inconclusive. For example, no significant cognitive improvements were observed following B-vitamin supplementation (Sultana et al., 2024). Moreover, excessive use of dietary supplements—particularly metal chelators or high-dose trace elements—may be detrimental by disrupting metal homeostasis or inducing toxicity. These results highlight the need for carefully designed supplementation strategies guided by reliable biomarkers and tailored to individual metabolic profiles.

## 2 Alzheimer’s disease and natural products

Herbal medicine (HM) is widely used in East Asia to treat cognitive diseases, including AD. Several herbal species, including Ginkgo biloba, Withania somnifera, Panax ginseng, Curcuma longa, and Camellia sinensis, possess bioactive phytochemicals with antioxidant, anti-inflammatory, anti-amyloidogenic, and neurotrophic activities important to AD therapy (Alzobaidi et al., 2021). Traditional Korean Medicine (TKM) and Traditional Chinese Medicine (TCM) relate memory loss and dementia to inadequacies in renal essence, blood flow stagnation, and the accumulation of pathogenic poisons (Youn et al., 2021; Wang et al., 2022a). A recent analysis of memory-enhancing herbal formulas in TKM highlights their mechanisms in promoting hippocampal neurogenesis, reducing oxidative stress, and modulating neuroinflammation (Zhang Y. et al., 2019; Lee et al., 2022).

### 2.1 Mechanistic evidence of natural compounds

Preclinical studies have demonstrated that these natural products modulate multiple AD-related pathways including reducing Aβ burden, inhibiting tau hyperphosphorylation, preserving mitochondrial function, and suppressing pro-inflammatory cytokines (Wang et al., 2022b). Systematic reviews and meta-analyses demonstrate that when combined with standard treatments, HM can improve cognitive performance, delay functional decline, and improve quality of life in Alzheimer’s patients.

### 2.2 Experimental models and multi target potentials

Natural products offer multi-targeted effects, making them well-suited for addressing the multifactorial pathology of AD. Experimental studies have utilized diverse animal models including transgenic mice (APP/PS1, 5×FAD), senescence-accelerated mice (SAMP8), and even *Drosophila melanogaster*—to explore these mechanisms. The ability of herbal compounds to restore redox homeostasis, enhance synaptic signaling, and inhibit apoptotic pathways underscores their therapeutic versatility.

### 2.3 Network pharmacology: a revolutionary tool in Alzheimer’s disease research

Network pharmacology is a systems-based approach that integrates bioinformatics, cheminformatics, and systems biology to investigate the complex interactions between drugs, targets, and diseases. Unlike traditional “one drug, one target” paradigms, network pharmacology enables identification of multiple molecular targets and pathways simultaneously (Li X. et al., 2023), making it ideal for dissecting the multifaceted nature of AD. It leverages large-scale databases such as TCMSP, STITCH, STRING, GeneCards, and OMIM to construct compound target pathway networks, helping researchers identify hub genes, key targets, and synergistic interactions. Network pharmacology enhances the precision of drug discovery by linking pharmacological targets to disease mechanisms through target prediction algorithms and functional enrichment analyses, such as Gene Ontology (GO) and Kyoto Encyclopedia of Genes and Genomes (KEGG) pathway mapping (Singh et al., 2022).

### 2.4 Application to AD: mapping complexity

In AD research, this approach uncovers how phytochemicals modulate core pathological pathways including Aβ aggregation, tau phosphorylation, oxidative stress, and neuroinflammation via shared molecular targets (Naseri et al., 2019). For instance, certain herbal compounds such as curcumin, resveratrol, and ginsenosides have been shown to interact with key nodes such as MAPK, AKT, BACE1, and TNF-α in AD networks.

### 2.5 Traditional herbal medicine meets network science

Network pharmacology is particularly suitable for analyzing traditional herbal formulas, which contain multiple components acting on diverse biological targets. By deconstructing herbal formulas into their constituent compounds and targets, this approach provides mechanistic insights into how multi-component therapies achieve synergistic efficacy. In TKM and TCM, such analyses have clarified the roles of specific herbs in modulating neuroinflammatory and redox-related signaling pathways. Several recent studies employing this methodology have uncovered that traditional memory-enhancing formulas regulate PI3K/AKT, Nrf2, MAPK, and NF-κB pathways key nodes in the AD signaling network (Fão et al., 2019).

#### 2.5.1 Multi-omics approaches and systems biology in Alzheimer’s research

**FIGURE 2:**
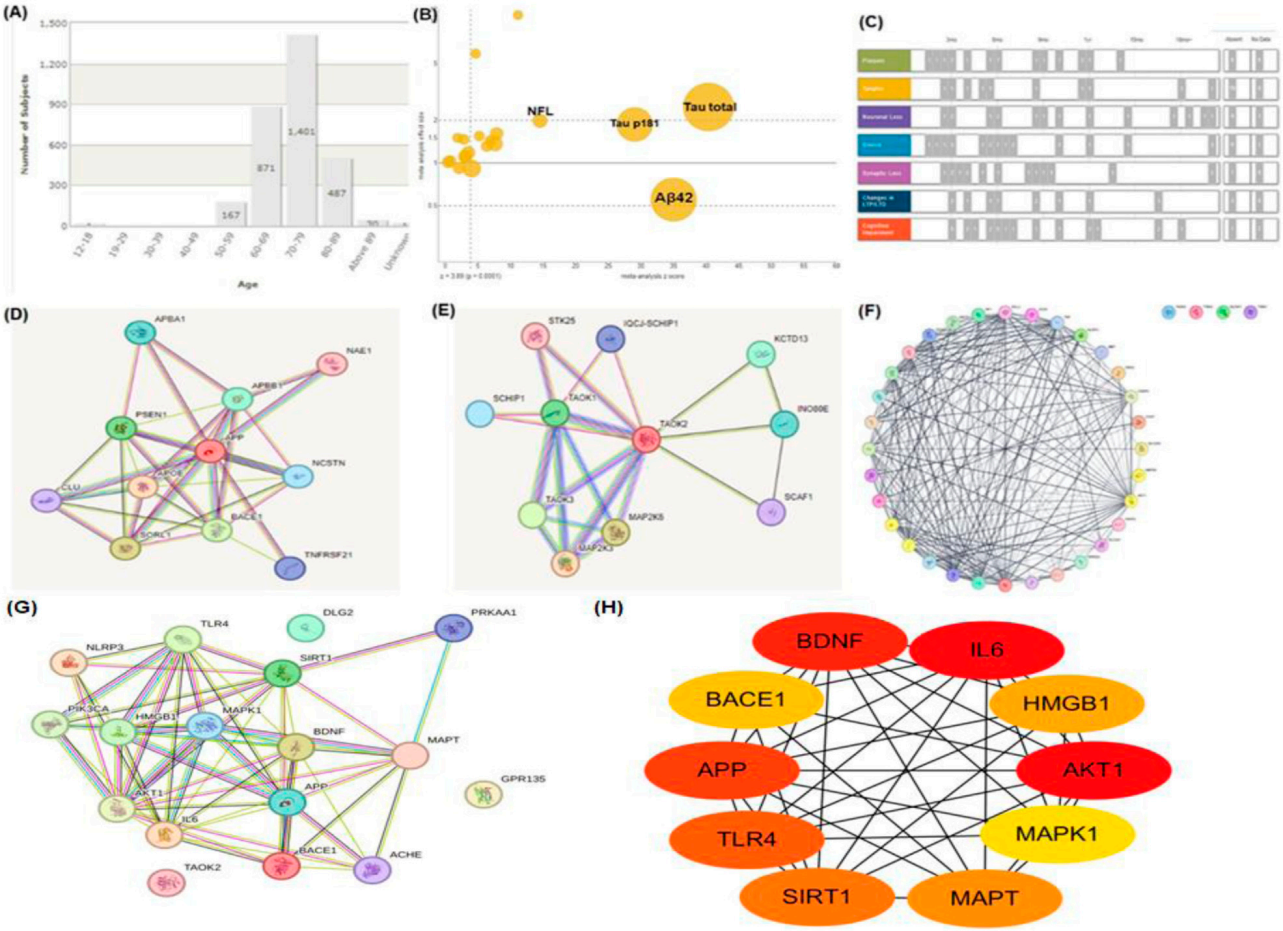
Bioinformatics Analysis and PPI Networks in Dementia Research. **(A)** Distribution of dementia incidence by age group. **(B)** Comparative expression levels of key genes, including tau and amyloid-beta 42 (Aβ42), between dementia patients and healthy individuals. **(C)** Overview of mutation types, their reported occurrences, and predicted mutation frequencies in dementia-related genes. **(D)** PPI network analysis of amyloid-beta 42 (Aβ42), showing its interactions with other proteins associated with dementia. **(E)** PPI network of tau protein, highlighting its connections to other dementia-related proteins. **(F)** Integrated PPI network showing combined interactions between Aβ42, tau, and other critical dementia-related proteins. **(G)** PPI network based on bioactive phytochemicals derived from medicinal plants studied in AD, highlighting their multi-target potential. **(H)** Top 10 hub proteins ranked by node degree in the phytochemical-target PPI network, representing key therapeutic targets.

Recent advances in multi-omics technologies—including transcriptomics, metabolomics, proteomics, and spatial transcriptomics—have opened new avenues for elucidating the molecular mechanisms through which natural products influence AD pathogenesis (Ma et al., 2024a). In contrast to single-layer analyses, multi-omics approaches integrate diverse biological data to provide a systems-level perspective on how phytochemicals interact with complex disease pathways (Zhu et al., 2022). For example, a combined transcriptomic and metabolomic analysis was used to investigate the effects of ginsenoside Rg1 in a rat model of AD (Ye et al., 2023). This integrative approach revealed that Rg1 modulates key metabolic pathways related to oxidative stress and energy balance, as well as genes involved in inflammation and synaptic signaling (Wu et al., 2022). In another study, systems pharmacology modeling predicted multi-target interactions of resveratrol in AD, highlighting its regulatory effects on autophagy-related genes and its involvement in the PI3K/Akt/mTOR signaling axis (Li Y. et al., 2023). These integrative omics-based approaches not only validate traditional ethnopharmacological knowledge but also facilitate target identification, drug repurposing, and biomarker discovery (Zhu et al., 2022). Collectively, bioinformatics-driven multi-omics platforms provide a powerful framework for delineating the multifaceted pharmacological actions of natural compounds and accelerating the development of precision medicine strategies for AD. While omics and systems biology approaches offer valuable molecular insights, linking these findings to clinical outcomes remains a significant challenge. Core AD biomarkers such as Aβ42, total tau, phosphorylated tau (p-tau), and neurofilament light chain (NFL) are commonly used to monitor disease progression and treatment efficacy. However, in clinical trials involving natural compounds such as curcumin, resveratrol, and ginsenosides, these biomarkers have shown variable predictive value. For example, Aβ42 levels tend to plateau in later disease stages, reducing their dynamic utility, while tau and NFL levels may be confounded by coexisting neurodegenerative conditions, as illustrated in Figure 2B. Moreover, the interpretation of biomarker responses is complicated by the poor bioavailability and limited blood–brain barrier penetration of many phytochemicals. Consequently, biomarker endpoints in studies of natural products must be rigorously standardized and critically evaluated. To enhance sensitivity, specificity, and translational relevance, future research should incorporate multimodal biomarker strategies—combining imaging, fluid-based, and multi-omics analyses.

### 2.6 Limitations and future outlook

While powerful, this approach relies heavily on database accuracy, and functional validation through *in vitro* or *in vivo* experimentation remains essential. Emerging integration with omics platforms including spatial transcriptomics and metabolomics will enable higher-resolution modeling of drug–target–disease interactions in AD. As shown in Figure 2A, which illustrates the age distribution of study participants. The highest representation in the age groups of 60–69 and 70–79 reflects the heightened vulnerability of these age brackets to AD. This demographic analysis provides critical insights into the at-risk population, ensuring research efforts are appropriately targeted toward these groups. Key biomarkers, including Aβ42, total tau, phosphorylated tau (p181), and neurofilament light chain (NFL), are pivotal in diagnosing and tracking disease progression. As demonstrated in Figure 2B, elevated levels of tau and NFL indicate neurodegeneration, while reduced Aβ42 correlates with amyloid plaque pathology. These biomarkers underscore their indispensable role in early diagnosis, disease progression monitoring, and as targets for therapeutic intervention. The schematic in Figure 2C captures the sequential progression of hallmark AD features, such as amyloid plaques, tau tangles, neuronal loss, gliosis, synaptic degeneration, and cognitive decline. This temporal representation highlights critical windows for therapeutic intervention, emphasizing the importance of early detection and treatment to mitigate irreversible damage. Figure 2D reveals the intricate network of proteins and genes such as APOE, APP, beta-secretase 1(BACE1), and PSEN1 involved in amyloid metabolism and plaque formation. The interconnectedness of these molecular players underscores the challenge of targeting AD at the molecular level, requiring a multifaceted approach to disrupt these networks effectively. Similarly, Figure 2E focuses on TAO kinase 2 (TAOK2), a protein closely linked to tau phosphorylation and microtubule stability. This highlights its role as a potential therapeutic target in mitigating tau-associated neurodegeneration. The dense connectivity visualized in Figure 2F emphasizes the interplay between various signaling pathways and molecular mechanisms underlying AD. This holistic perspective is critical for the development of multi-targeted therapeutic strategies, as single-pathway interventions are unlikely to address the multifactorial nature of the disease. This analysis of AD through network pharmacology provides a promising roadmap for addressing the disease’s multifaceted pathology, paving the way for innovative, multi-targeted therapeutic solutions (Figure 2). To further enhance the interpretation of the combined PPI network (Figure 2F), we constructed an additional phytochemical-target-based PPI network (Figure 2G) using STRING analysis. The top 10 hub proteins ranked by node degree are summarized in (Figure 2H), highlighting critical intervention points in AD-related networks.

### 2.7 Pathogenesis of Alzheimer’s disease

**FIGURE 3:**
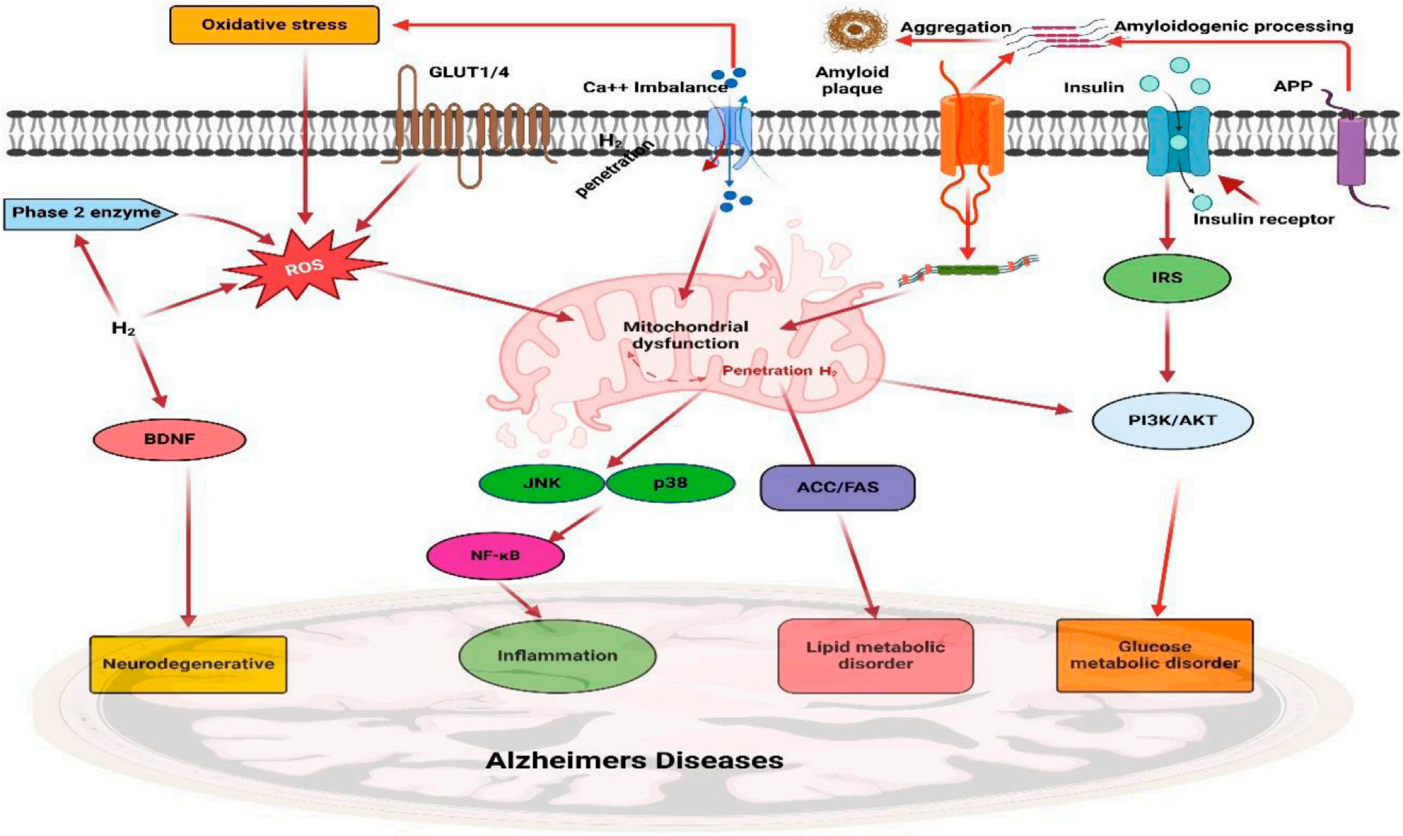
The pathophysiological molecular pathways of Alzheimer’s disease (AD). The intricate interactions between key pathways in AD, such as the amyloid route, tau pathology, oxidative stress, neuroinflammation, and metabolic dysfunction, are illustrated in this diagram. Together, these pathways result in synapse loss, neuronal death, and cognitive impairment, underscoring the need for multi-targeted AD treatment initiatives.

AD is driven by a complex interplay of genetic, molecular, and environmental factors, resulting in progressive neurodegeneration and cognitive impairment (Yuksel and Tacal, 2019). The key pathological hallmarks of AD include amyloid-beta (Aβ) plaque accumulation, neurofibrillary tangles of hyperphosphorylated tau, chronic neuroinflammation, oxidative stress, and mitochondrial dysfunction (Lv et al., 2020). In AD, tau becomes hyperphosphorylated and dissociates from microtubules, resulting in the formation of intracellular neurofibrillary tangles (Pavani and Tiwari, 2024). This disrupts axonal transport, destabilizes cytoskeletal architecture, and contributes to neuronal death (Duggal and Mehan, 2019). Recent findings suggest that pathological tau can spread trans-synaptically, accelerating neurodegeneration in a prion-like fashion (Meng et al., 2023). Microglial activation is a hallmark of AD, initially serving a protective role but becoming detrimental upon chronic stimulation (Li et al., 2021). Sustained microglial activation leads to the release of pro-inflammatory cytokines such as TNF-α, IL-1β, and IL-6, which exacerbate neuronal injury and amplify amyloid and tau pathology (Kaur et al., 2019). Oxidative stress, resulting from excessive reactive oxygen species (ROS) production and impaired antioxidant defense, plays a central role in AD progression (Yin, 2023). ROS cause lipid peroxidation, protein oxidation, and mitochondrial DNA damage, leading to neuronal apoptosis and synaptic dysfunction (Bhatia and Sharma, 2021). Other contributing factors include dysregulated calcium homeostasis (Buccellato et al., 2021). Mitochondria in AD show impaired electron transport chain activity, altered calcium buffering, and increased ROS generation (Simunkova et al., 2019). Mitochondrial dysfunction contributes to energy failure, triggers intrinsic apoptosis, and perpetuates oxidative stress, forming a self-reinforcing neurodegenerative cycle (Wang D. et al., 2021). Impaired mitophagy and fragmented mitochondrial morphology are emerging as novel contributors to neuronal vulnerability in AD (Cao et al., 2019). AD pathogenesis involves a number of interrelated pathways, including amyloid plaque development, tau protein hyperphosphorylation, oxidative stress, neuroinflammation, and metabolic failure (Figure 3). These degenerative processes all contribute to synapse loss, neuronal death, and cognitive decline, emphasizing the need for multi targeted treatment approaches.

Several natural compounds have been shown to modulate key signaling pathways implicated in the pathogenesis of AD. Curcumin inhibits the MAPK pathway by reducing ERK1/2 phosphorylation, thereby attenuating oxidative stress and neuronal apoptosis. Additionally, it suppresses NF-κB activation by preventing IκBα degradation and the nuclear translocation of the p65 subunit, resulting in reduced expression of pro-inflammatory cytokines. Resveratrol exerts anti-inflammatory and neuroprotective effects by activating SIRT1, which in turn suppresses NF-κB signaling. Epigallocatechin gallate (EGCG), a major polyphenol in green tea, mitigates amyloid-beta production by inhibiting β-secretase (BACE1) activity. Furthermore, EGCG enhances Nrf2-mediated antioxidant responses, thereby protecting against mitochondrial dysfunction. Collectively, these pathway-specific actions highlight the therapeutic promise of natural compounds as multi-targeted agents in AD treatment. A summary of these compounds, their associated molecular targets, and supporting references is provided in Table 1.

**TABLE 1:** The targeted molecular pathways of natural products in Alzheimer’s disease.

Natural product	Targeted pathways	Mechanism of action	References
Curcumin	MAPK/NF-κB	↓ERK1/2 phosphorylation, neuronal apoptosis, oxidative stress, IκBα degradation, p65 nuclear translocation, inflammation	Lv et al. (2024)
Resveratrol	SIRT1/NF-κB	↑SIRT1, ↓NF-κB, neuroinflammation	Peng et al. (2022)
EGCG	MAPK, NF-κB, SIRT1, Nrf2 pathway	↑Nrf2-mediated antioxidant response, ↓BACE1	Shah et al. (2024)
Ginsenosides	PI3K/Akt	↑PI3K/Akt signaling, neuronal survival	Zarneshan et al. (2022)
Quercetin	JNK/NF-κB	↓JNK activation, NF-κB, oxidative stress, inflammation	Tang et al. (2023)

MAPK, Mitogen-Activated Protein Kinase; NF-κB, Nuclear Factor kappa-light-chain-enhancer of activated B cells; SIRT1, Sirtuin 1; Nrf2, Nuclear factor erythroid 2–related factor 2; PI3K, Phosphoinositide 3-Kinase; Akt, Protein Kinase B; JNK, c-Jun N-terminal Kinase; EGCG, Epigallocatechin-3-gallate; ERK1/2, Extracellular Signal-Regulated Kinases 1 and 2; IκBα, Inhibitor of kappa B alpha; p65, RelA subunit of NF-κB; BACE1, Beta-site Amyloid Precursor Protein Cleaving Enzyme 1.

### 2.8 The impact of natural products on Alzheimer’s disease

AD, as a multifaceted neurodegenerative disorder, involves a complex array of pathological processes, including amyloid-beta (Aβ) plaque formation, tau protein hyperphosphorylation, oxidative stress, mitochondrial dysfunction, and chronic neuroinflammation. Given the limitations of current single-target therapies, interest has grown in natural products as promising multi-target agents for modulating AD pathology (Zhang et al., 2024). Natural compounds sourced from plants, fungi, and marine organisms contain diverse bioactives capable of interacting with multiple molecular targets implicated in AD pathogenesis (Nahar et al., 2025). These compounds exhibit antioxidant activity, inhibit amyloid and tau aggregation, modulate neuroinflammation, protect mitochondrial function, and promote neuronal survival. This broad-spectrum activity makes natural products well-suited to address the multifactorial nature of AD (Chen et al., 2021). Curcumin (from *Curcuma longa*), a potent antioxidant and anti-inflammatory agent, curcumin disrupts Aβ aggregation, dissolves pre-formed amyloid fibrils, and protects neurons from oxidative damage (Yang et al., 2005; Doytchinova et al., 2020). Resveratrol (from grapes and red wine) enhances mitochondrial function, reduces oxidative stress, and activates neuroprotective proteins like SIRT1 (Zhou J. et al., 2021). *It also facilitates Aβ clearance via autophagy* (Kou and Chen, 2017). Huperzine A (from *Huperzia serrata*), an acetylcholinesterase (AChE) inhibitor that enhances memory by increasing acetylcholine (ACh) level shown to improve memory and inhibit glutamate-induced excitotoxicity (Qian and Ke, 2014; Lee et al., 2018). Ginkgo biloba terpenoids improve cerebral blood flow, reduce oxidative stress, inhibit Aβ aggregation, and regulate microglial activity to curb neuroinflammation (Xia et al., 2024). Ginsenosides (from *Panax ginseng*) exhibit neuroprotective effects by reducing oxidative stress, enhancing mitochondrial function, and preventing tau hyperphosphorylation (Shi et al., 2022; Zhang et al., 2023). EGCG (a catechin from green tea) prevents Aβ aggregation, reduces plaque toxicity, and enhances synaptic plasticity while providing strong antioxidant effects (Chen et al., 2020). In addition, marine-derived compounds such as fucoidans and bryostatin (Bălaşa et al., 2020), along with cannabinoids from cannabis, have shown potential for neuroprotection, inflammation reduction, and synaptic repair (Valeri and Mazzon, 2021). To help illustrate the therapeutic significance and developmental advancement of these natural chemicals, Table 2 highlights their molecular targets, experimental models used, clinical status, and potential future research avenues.

**TABLE 2:** Summary of natural compounds assessed for Alzheimer’s disease.

Compound	Model used	Molecular target	Clinical status	Future direction	References
Curcumin	*In vitro*, *In vivo*	Aβ aggregation, oxidative stress	Phase II trials	Increase bioavailability; test in combination with AD medications	Azzini et al. (2024)
Resveratrol	*In vitro* Clinical	SIRT1 activation, mitochondrial protection	On going	Verify long-term effectiveness; research dose optimization	Brown et al. (2024)
Huperzine A	*In vivo* *In vitro* Clinical	Inhibition of acetylcholinesterase	Approved in China; testing conducted elsewhere	Analyze combination therapy; and multi-target potential	Shukla et al. (2022)
Ginkgo biloba	*In vivo* *In vitro* Clinical	Antioxidant, anti-inflammatory and neuroprotective	widely utilized; inconsistent trial outcomes	Stratify patient response; investigate synergistic combinations	Akanchise and Angelova, (2023)
Ginsenosides	*In vitro*, *In vivo*	Tau, mitochondrial malfunction, and oxidative stress	Initial clinical trials	Conduct large-scale RCTs; evaluate delivery systems	Chen et al. (2024)
EGCG	*In vitro*, *In vivo*	Aβ aggregation, oxidative stress	Preclinical trial	Investigate BBB permeability; evaluate synergistic potential	Rassu et al. (2023)

RCTs, Randomized controlled trials; EGCG, (−)-Epigallocatechin gallate.

However, despite the widespread use of *in vitro* and *in vivo* models—such as transgenic animal models and brain organoids—to investigate the effects of natural compounds, these systems often fall short in capturing the full complexity of late-onset Alzheimer’s disease (LOAD). In particular, they frequently fail to account for age-associated changes, comorbidities, and human-specific pathological features (Vitek et al., 2020). These limitations may partially explain the discrepancy between promising preclinical results and the limited success observed in clinical trials. To bridge this translational gap, future research should incorporate advanced platforms, including patient-derived induced pluripotent stem cell (iPSC) models, aged brain organoids, and computational models of disease progression, to more accurately assess the therapeutic potential of natural products.

Moreover, although many natural compounds share neuroprotective mechanisms—such as antioxidant and anti-inflammatory effects—their actions are not necessarily redundant (Zhang et al., 2022). The pharmacological efficacy of each compound may vary significantly depending on factors such as bioavailability, target specificity, and downstream signaling pathways. Considering the clinical differences between early-onset Alzheimer’s disease (EOAD) and LOAD, future investigations should explore how these compounds interact with subtype-specific pathological processes. Advanced disease models, including genetically stratified organoids, patient-derived iPSC lines, and age-appropriate 3D brain cultures, may offer more refined platforms to dissect these differences and identify compound-subtype associations (Sirkis et al., 2022).

Additionally, growing evidence suggests that the therapeutic efficacy of natural products in AD may be modulated by genetic factors, particularly the apolipoprotein E (APOE) genotype (Frisoni et al., 2022). APOE4 carriers, who exhibit greater susceptibility to AD, display distinct oxidative stress and inflammatory profiles that may influence their responsiveness to phytochemicals (Sharifi-Rad et al., 2022). For instance, curcumin has been shown to reduce amyloid plaque burden more effectively in APOE3 than in APOE4 transgenic mouse models (Far et al., 2024). While genotype-specific responses remain incompletely understood, recent clinical trials have demonstrated that resveratrol exerts neuroprotective effects by mitigating neuroinflammation, enhancing mitochondrial function, and preserving blood-brain barrier integrity in patients with mild cognitive impairment (MCI) and early AD (Buglio et al., 2022). These findings underscore the importance of incorporating genetic stratification into precision medicine approaches that utilize natural compounds for AD treatment.

### 2.9 Natural compounds frequently function via numerous mechanisms

Natural products exert therapeutic effects in AD through multiple interrelated mechanisms. Several natural compounds reduce Aβ toxicity by inhibiting its aggregation, promoting its clearance via autophagy, and limiting synaptic dysfunction (Zeng et al., 2019). Antioxidant-rich compounds reduce ROS production, protecting neurons from oxidative stress-induced apoptosis (Simunkova et al., 2019). Many phytochemicals inhibit glial activation and attenuate neuroinflammatory signaling cascades (Deng et al. 2023). AChE inhibitors, such as huperzine A, increase ACh levels to support memory and cognitive function (Friedli and Inestrosa, 2021). Compounds like resveratrol improve mitochondrial bioenergetics, enhance ATP production, and reduce ROS generation (Moawad et al., 2024). However, clinical translation is hindered by low oral bioavailability, limited BBB permeability, and inconsistent pharmacokinetics. To overcome these limitations, researchers are exploring nanoformulations, targeted delivery platforms, and prodrug strategies. Integrating *in silico* modeling, cell-based assays, and animal studies allows for a comprehensive understanding of how natural compounds act in AD. Multimodal research is essential to bridge the translational gap and optimize therapeutic development pipelines. Their multi-target pharmacology presents a unique opportunity for breakthrough therapies in complex disorders such as AD.

### 2.10 *In vitro* studies

*In vitro* models are instrumental in unraveling the complexities of AD, providing a controlled environment to investigate cellular and molecular mechanisms. They serve as efficient platforms to evaluate the effects of natural products in a reproducible and cost-effective way (Vignon A. et al., 2021). Traditional two-dimensional (2D) cell cultures have long been the cornerstone of AD research (Guido et al., 2023). However, 2D cultures oversimplify the cellular architecture and fail to capture the complex brain microenvironment (Acharya et al., 2024). Recent advancements, such as three-dimensional (3D) brain organoids derived from induced pluripotent stem cells (iPSCs), have significantly enhanced the physiological relevance of *in vitro* models (Zivko et al., 2024). These systems reproduce key hallmarks of AD pathology, including Aβ deposition, tau pathology, and glial activation. This advancement narrows the gap between reductionist models and physiological relevance, improving preclinical assessment of natural products (Lei et al., 2024). Several studies have demonstrated the efficacy of natural products in targeting AD-related mechanisms using *in vitro* models (Table 3).

**TABLE 3:** Summary of in vitro studies in AD research.

Classification	Compound/Extract	Source	Experimental model	Dose; duration	Efficacy	Mechanism	References
Plant	Fenugreek Water Extract	Trigonella foenum-graecum	PC-12 cells	10 μg/mL	Antioxidant, anti-amyloid	↑BDNF, ↓ROS	Brimson et al. (2022)
Plant	Salvia fruticosa water extract	Salvia fruticosa	SH-SY5Y (neuroblastoma) cell	250 and 100 μg/mL	Anticholinesterase, antioxidant	↓GSK-3β, CK-1δ, BACE-1, ROS	Gürbüz et al. (2021)
Plant	Shaoyao Gancao Tang extract	Shaoyao Gancao Tang	Aβ-GFP SH-SY5Y		Anti-inflammatory, anti-amyloid	↓iNOS, NLRP1, TNF-α, IL-1β, IL-6, ROS	Chiu et al. (2021)

BDNF, Brain-derived neurotrophic factor; TNF-α, tumor necrosis factor-alpha; IL-1β, interleukin-1β; MCP-1, Monocyte chemoattractant protein-1; MIP-1b, Macrophage inflammatory protein-1beta; IL-8, Interleukin-8; GSK-3β, Glycogen Synthase Kinase-3β; CK-1δ, Casien Kinase-1δ; Bace-1, β-secretase; iNOS, inducible nitric oxide synthase; NLRP1, NLR, family pyrin domain containing 1; NLRP3, NLR, family pyrin domain containing 3; ROS, reactive oxygen species; Hsp70s, 70 kDa heat shock proteins.

#### 2.10.1 Limitation of *in-vitro* model

Despite their value in mechanistic research, *in vitro* models present several critical limitations that constrain their translational applicability (Blanchard et al., 2022). Conventional 2D cell cultures oversimplify the complex cytoarchitecture, cell cell signaling, and dynamic microenvironment of the human brain (Anwar et al., 2022). Critical elements such as the extracellular matrix (ECM) are poorly represented, limiting realistic cellular organization and response (Cenini et al., 2021). Cell lines often have limited proliferative capacity and do not recapitulate chronic disease processes such as progressive amyloid deposition and tau aggregation (Slanzi et al., 2020). Cultured cells have limited lifespans, restricting long-term investigations into processes like amyloid plaque accumulation, tau tangle formation, and progressive neuronal degeneration (Armijo et al., 2021). Extended culturing can induce genomic instability, including chromosomal aberrations and copy number variations, which compromise reproducibility and relevance (Rodriguez-Jimenez et al., 2023). *In vitro* systems also fail to reproduce essential features such as immune neural interactions, BBB dynamics, and pharmacokinetic behaviors (Bukhari, 2022). Emerging technologies, including brain organoids and microfluidic “organ-on-chip” platforms, provide enhanced physiological relevance by simulating multicellular brain environments and nutrient flow (Castiglione et al., 2022). Nevertheless, these models remain technically challenging, costly, and require standardization for broader application. Thus, *in vitro* studies should be integrated with *in vivo* approaches to provide a more comprehensive understanding of AD pathology and therapeutic efficacy. Animal models contribute systemic context enabling evaluation of cognitive outcomes, neuroimmune responses, and long-term progression which are beyond the scope of cell-based systems.

### 2.11 *In vivo* studies

*In vivo* studies are pivotal in bridging the gap between preclinical findings and clinical applications for AD. They offer a physiologically integrated setting to evaluate disease mechanisms and therapeutic efficacy of natural products. These models capture dynamic interactions between brain regions, immune cells, and systemic physiology features not replicable *in vitro*. Genetically engineered animal models, particularly mice, have revolutionized AD research. Humanized models expressing risk genes like APOE4 faithfully recapitulate genetic susceptibility and clinical phenotypes observed in human AD (Vitek et al., 2020). Advanced techniques like CRISPR gene-editing technology have further enhanced the precision of *in vivo* models, allowing the replication of specific mutations linked to AD (Bhardwaj et al., 2022). These innovations facilitate the study of epigenetic modifications, combinatorial gene interactions, and environmental triggers in AD (Zeiss, 2020). Non-human primates (NHPs), such as rhesus monkeys, naturally exhibit age related tau and amyloid β (Aβ) pathologies, providing models that closely mimic human disease progression (Li et al., 2019). Due to their high translational value, NHPs help unravel complex immune and neurodegenerative mechanisms relevant to therapeutic modulation (Fulop et al., 2021). Recent technological advances have enhanced the translational relevance of animal models. Techniques like PET and twophoton microscopy allow longitudinal monitoring of pathological progression in living animals. *Biomarker studies, involving cerebrospinal fluid (CSF) and blood analyses, enhance the translational relevance of these models by paralleling findings observed in human AD patients* (Bjorkli et al., 2020). Behavioral paradigms such as Morris water maze and Y-maze offer quantifiable endpoints (Okada et al., 2021), that correlate with histopathological and molecular findings (Shokhirev and Johnson, 2022). Environmental and lifestyle model (Decourt et al., 2022). such as chronic stress or exposure to metals like aluminum (Gom, 2024), worsen AD features via oxidative injury and proteinopathy enhancement (Althobaiti, 2024). Rodents remain widely used due to practical advantages such as short lifespans, established behavioral assays, and genetic modifiability. Popular rodent strains include APP/PS1, 3×Tg-AD, 5×FAD, SAMP8, BALB/c, ICR, and C57BL/6 mice, as well as Sprague Dawley and Wistar rats. They have been extensively used to evaluate the multi-target effects of natural compounds on amyloidogenesis, tauopathy, mitochondrial health, and glial reactivity (Dhapola et al., 2023).

#### 2.11.1 Therapeutic investigations

Studies utilizing *in vivo* models have demonstrated the therapeutic efficacy of various natural compounds in targeting AD elated pathologies (Table 4). Fenugreek Water Extract (FWE) reduced Aβ levels, improved cognitive performance, and decreased oxidative stress in APP/PS1 mice. Increased antioxidant markers (GSH, SOD), and downregulated pro-inflammatory cytokines (IL-6, TNF-α, IBA-1, GFAP (Liu et al., 2018). Banxia Xiexin Decoction (BXD) improved memory and spatial learning in APPswe/PS1dE9 mice. Restored PI3K/Akt signaling and increased GLUT1/GLUT3 expression (Chen et al., 2018). Huanglian Jiedu Decoction (HLJDD), (cognitive deficits and reduced Aβ accumulation and modulated gut dysbiosis) *ameliorated memory deficits, reduced amyloid burden, improved gut microbiota composition,* reduced pro-inflammatory markers (IL-6, IL-1β), and enhanced antioxidative enzymes (SOD, IL-4) (Gu et al., 2021). haoyao Gancao Tang (SG-Tang) reduced Aβ aggregation, tau hyperphosphorylation, and neuroinflammation in 3×Tg-AD mice. Suppressed inflammatory pathways (NLRP1, NLRP3) and oxidative stress (Chiu et al., 2021).

**TABLE 4:** Key findings from in vivo studies with APP transgenic mice.

Classification	Compound/Extract	Source	Experimental Model	Dose; Duration	Efficacy	Mechanism	References
Plant	Fenugreek Water Extract	Trigonella foenum-graecum	APP/PSI transgenic mice	50, 500 mg/kg; 4 weeks	Antioxidant, anti-inflammatory	↑GSH, SOD; ↓IL-6, TNF-α, IBA-1, GFA	Liu et al. (2018)
Plant	Banxia Xiexin Decoction	Traditional Formula	APPswe/PS1dE9 mice	650 mg/kg/day	Improved memory, restored synaptic functions	↑GLUT 1, GLUT 3, P13K, Akt	Chen et al. (2018)
Plant	Huanglian jiedu decoction	Traditional Formula	APP/PS1 mice	3 months	Ameliorated cognitive deficits, modulated gut microbiota	↑IL-4, IL-10; ↓Aβ, IL-6, MCP-1	Gu et al. (2021)
Plant	Shaoyao Gancao Tang	Traditional Formula	APP/PS1/Tau mice		Reduced Aβ aggregation, tau hyperphosphorylation	↓NLRP1, NLRP3	Chiu et al. (2021)
Plant	Shexiang Baoxin Pill	Traditional Formula	APP/PS1 mice	172, 344 mg/kg/day; 4 months	Anti-apoptotic, anti-inflammatory	↑Bcl-2; ↓NOS, IL-6, TNF-α	Hu et al. (2020)

GSSG, glutathione disulfide; GSH, glutathione; SOD, superoxide dismutase; GLUT, glucose transporter; Akt, Protein kiase B; IDE, Insulin-degrading enzyme; NLRP1, NLR, family pyrin domain containing 1; NLRP3, NLR, family pyrin domain containing 3; SOD, superoxide dismutase; MDA, malonic acid; COX-2, Cyclooxygenase 2; 5-LOX, 5-Lipoxygenase; MCP-1, Monocyte chemotactic protein 1; DCA, deoxycholic acid; T-α-MCA, Tauro-α-muricholic acid; THDCA, taurohydeoxycholic acid; NO, nitric oxide; NOS, nitric oxide synthase; AChE, acethlcholinesterase; PS1, Presenilin 1; ADAM, α-secretase.

### 2.12 BALB/c mice in Alzheimer’s disease research

BALB/c mice have emerged as a valuable model in AD research due to their TH2-biased immune responses and genetically stable background. This unique immune profile supports enhanced antibody production and modulates neuroinflammation, a critical factor in AD pathology (Table 5) (Cacabelos, 2020). BALB/c mice provide essential insights into both immunological and neurodegenerative mechanisms, contributing significantly to understanding and addressing AD-related challenges. One hallmark of AD is the activation of microglia, the brain’s resident immune cells, which can either clear amyloid-β (Aβ) plaques or exacerbate neuronal damage through chronic inflammation (Wang J. M. et al., 2017; Edler et al., 2021). BALB/c mice offer a unique platform for studying how TH2-mediated immune responses influence microglial activity, which can modulate neuronal health and disease outcomes (Mukhopadhyay et al., 2020). Although BALB/c mice do not naturally develop AD (Mancuso et al., 2019), their genetically stable background makes them an excellent model for isolating the effects of genetic modifications, environmental factors, or therapeutic interventions on AD pathology (Balietti et al., 2023). Through targeted genetic engineering, researchers can replicate key AD features, such as tau tangles and amyloid plaque deposition, allowing for precise investigation of the disease mechanisms. The TH2-skewed immune environment in BALB/c mice promotes the production of anti-inflammatory cytokines like IL-4 and IL-10 while reducing cytotoxic T-cell responses (Alvarez-Sanchez and Dunn, 2022; Kumar et al., 2023). This profile creates opportunities to study how anti-inflammatory pathwa ys interact with neuroinflammation, amyloid accumulation, and tau pathology in AD. Such investigations are critical for understanding the intricate balance of cytokine-mediated neuroinflammation and its influence on AD progression (Xavier et al., 2023). Behaviorally, BALB/c mice display elevated anxiety and reduced social interaction compared to other strains, traits that mirror emotional (Mehla et al., 2022) and behavioral changes often observed in Alzheimer’s patients, such as anxiety, impaired social interaction, and disinhibition (Mitrea et al., 2022). This makes BALB/c mice particularly effective for studying the emotional and behavioral dimensions of AD. By leveraging their immune characteristics, genetic stability, and behavioral traits, BALB/c mice provide a versatile and powerful tool for AD research. They enable a deeper understanding of the interplay between immune responses, neuroinflammation, and genetic modifications, paving the way for targeted and innovative therapeutic strategies.

**TABLE 5:** *In vivo* Studies Using BALB/c Mice.

Classification	Compound/Extract	Source	Experimental Model	Dose; Duration	Efficacy	Mechanism	References
plant	Bacopa floribunda	Saponins flavonoid	BALB/c mice	saponins (100 mg/kg and 200 mg/kg) and flavonoid (100 mg/kg)	Anti-oxidant Anti-inflammatory effects	↓ MDA, IL1β, TNF-α	Oyeleke and Owoyele, (2022)
plant	Ershiwuwei Shanhu Pills (ESP)		BALB/c mice	Low [200 mg kg∼ (−1)], medium [400 mg kg∼ (−1)] and high (800 mg kg∼ (−1)	Anti-AD	Akt/mTOR/GSK-3β	Luo et al. (2022)
plant	Lycium barbarum water extract	*Lycium barbarum*	BALB/c mice	0.5, 2.0 g/kg 4 weeks	Improved endurance, reduced apoptosis	↑ACh, ChAT	Hu et al. (2018)

Bcl-2, Apoptosis regulator Bcl-2; Bax, Apoptosis regulator BAX; ach, Acetylcholine; ChAT, choline acetyltransferase; c-capase-3, -8, -9, Cleaved capase-3, -8, -9, Malondialdehyde (MDA).

#### 2.12.1 Therapeutic investigations

BALB/c mice have served as an invaluable model for investigating the therapeutic potential of natural compounds in AD research. Bacopa floribunda (BF), a locally available plant in Southwestern Nigeria, is traditionally revered as a memory enhancer and brain tonic in both traditional and Ayurvedic medicine. BF has been employed to combat aging, enhance memory, and prevent psychological disorders. Despite its historical usage, the precise mechanisms of action of BF’s bioactive phytochemicals, particularly in relation to dementia, remain insufficiently explored. Further scientific studies are imperative to elucidate its therapeutic potential and underlying molecular pathways (Oyeleke and Owoyele, 2022). The Tibetan patent medicine Ershiwuwei Shanhu Pills (ESP) has shown significant potential in alleviating AD symptoms in BALB/c mice. ESP demonstrated a capacity to improve learning and memory deficits, as well as oxidative damage, in AD models induced by D-galactose and aluminum chloride. These effects were achieved through the regulation of the Akt/mTOR/GSK-3β signaling pathway, positioning ESP as a promising therapeutic agent for addressing cognitive decline associated with AD (Luo et al., 2022). The water extract of *Lycium barbarum* (LB), administered at doses of 0.5 g/kg and 2.0 g/kg for 4 weeks, significantly improved behavioral and cognitive performance in BALB/c mice. LB enhanced endurance, increased horizontal and vertical movement, and reduced escape latency. These effects were attributed to elevated ACh and choline acetyltransferase (ChAT) levels in the serum and hypothalamus, promoting better neuronal communication and reduced apoptosis (Hu et al., 2018).

### 2.13 SAMP8 mice and 5×FAD mice insights in Alzheimer’s disease research

The identification of mutations linked to familial AD has provided critical insights into the fundamental mechanisms underlying disease pathogenesis and progression. These discoveries have enabled the development of animal models, which play a pivotal role in unraveling molecular pathways and advancing therapeutic interventions for AD (Lloyd et al., 2020). The 5×FAD and SAMP8 mouse models are among the most widely used in AD research, offering valuable insights into disease mechanisms, progression, and potential therapeutic interventions. Both models replicate critical hallmarks of AD, including amyloid-β plaque deposition, neuroinflammation, oxidative stress, and cognitive decline, making them indispensable tools for preclinical studies.

#### 2.13.1 APP (amyloid precursor protein) mutations

##### 2.13.1.1 K670N/M671L (Swedish mutation)

Cystatin C, a critical secretory cofactor for neurogenesis, possesses strong protease inhibitor activity and plays a significant role in neurological health. Genetic polymorphisms of cystatin C are associated with AD, while the L68Q mutation leads to hereditary cerebral hemorrhage with amyloidosis of the Icelandic type, where cystatin C and β-amyloid co-deposit in cortical blood vessels. To explore whether cystatin C and β-amyloid also co-localize in brain amyloid plaques, researchers analyzed transgenic mice carrying the Swedish APP (SweAPP) mutation, leading to elevated Aβ production and early plaque deposition (Steinhoff et al., 2001; Ilievski et al., 2018). The APPSwe-neuron-specific enolase (NSE) mouse model expresses human APP695 with the KM670/671NL mutation under the NSE promoter. Histological analysis revealed widespread, intensive Aβ42 staining in neurons of the cortex and hippocampus at 12 months, though no amyloid plaques were detected (Hwang et al., 2004). The Tg2576 mouse model also carries human APP695 with the KM670/671NL mutation, driven by the hamster prion protein promoter. Histological studies showed amyloid plaques in the cortex and hippocampus beginning at 11 months, without evidence of NFTs. Microglial activation was observed at 10 months, but no neuronal loss was noted. However, dendritic spine loss in the CA1 region of the hippocampus appeared as early as 4.5 months, with memory deficits becoming evident at 9 months (Hsiao et al., 1996; Lanz et al., 2003). These models highlight distinct pathological and temporal features of AD, including early synaptic changes and amyloid accumulation, providing valuable tools for studying disease progression and potential therapeutic targets.

##### 2.13.1.2 E693del (Osaka) mutation

The APP E693Δ-Tg (Osaka) mouse model, which carries the Osaka mutation (APP695) under the control of the mouse prion promoter, expressed comparable levels of mutant human APP and endogenous mouse APP. Histological analysis revealed no neurofibrillary tangles (NFTs), but abnormal tau phosphorylation was observed at 8 months. Intraneuronal Aβ accumulation was detected in the hippocampus and cerebral cortex at 8 months, without amyloid plaque formation. Microglial activation occurred at 12 months, astrocyte activation at 18 months, and neuronal loss and synaptic loss in the CA3 region of the hippocampus were evident by 18 and 8 months, respectively. Memory deficits were detected at 8 months (Tomiyama et al., 2010; Umeda et al., 2011). The OSK-KI mouse model, generated by knocking the Osaka mutation into the endogenous mouse APP gene, displayed APP expression levels similar to those of wild-type (WT) mice. Homozygous mice showed abnormal tau phosphorylation and intraneuronal Aβ accumulation in the hippocampus and cerebral cortex at 8 months, with heterozygotes exhibiting slight Aβ accumulation at 24 months. In homozygotes, microglial and astrocyte activation appeared at 12 months, neuronal loss in the hippocampus and entorhinal cortex at 24 months, and synaptic loss at 8 months. In contrast, heterozygotes exhibited gliosis and synaptic loss only at 24 months. Memory deficits in homozygotes were evident as early as 4 months (Umeda et al., 2017). These models demonstrate the distinct temporal and pathological progression of AD ike features, including tau abnormalities, intraneuronal Aβ accumulation, and synaptic and neuronal loss, making them valuable tools for studying disease mechanisms and therapeutic strategies.

##### 2.13.1.3 E693G (Arctic) mutation

Cellular studies of APP processing revealed significant changes in Aβ dynamics. Total secreted Aβ38 levels were elevated compared to WT controls, while Aβ40 levels remained unchanged. Aβ42 levels and the Aβ42/Aβ40 ratio were reduced, but polymerization of Aβ40 and Aβ42 increased, along with enhanced resistance to proteolytic degradation by neprilysin (Nilsberth et al., 2001; Murakami et al., 2002; Tsubuki et al., 2003; Yamamoto et al., 2004). The TgAPParc mouse model, which carries the E693G mutation (APP695) under the control of the murine Thy1.2 promoter, exhibited 3- to 7-fold higher mutant human APP expression compared to endogenous mouse APP. Histological studies revealed no NFTs. However, strong intracellular Aβ immunoreactivity was detected in the hippocampus and cortex at 3 months. Diffuse extracellular Aβ immunoreactivity appeared in some brain regions by 4 months, plaque-like structures in the subiculum by 6 months, and dense Aβ plaques with Congo red birefringence in the subiculum by 9 months. Biochemical analyses confirmed increased levels of Aβ40 and Aβ42 at 12 months, and memory deficits were observed by 15 months (Rönnbäck et al., 2011; Rönnbäck et al., 2012). These findings underscore the unique pathological timeline and molecular changes in the TgAPParc model, providing a valuable tool for studying Alzheimer’s disease pathogenesis and therapeutic development.

##### 2.13.1.4 E693Q (Dutch) mutation

Studies of APP processing in cellular models revealed distinct alterations compared to WT controls. Total secreted Aβ and Aβ38 levels remained unchanged, while secreted Aβ40 and Aβ42 levels decreased, accompanied by a reduced Aβ42/Aβ40 ratio. Aβ40 polymerization was enhanced, and resistance to proteolytic degradation by neprilysin increased (Tsubuki et al., 2003; Yamamoto et al., 2004). The APP Dutch mouse model, carrying the E693Q mutation (APP751) under the murine Thy1 promoter, exhibited no neurofibrillary tangles (NFTs) or amyloid plaques. However, cerebral amyloid angiopathy (CAA) developed at 22 months, followed by microglial and astrocyte activation and cerebral hemorrhage at 29 months (Herzig et al., 2004). A second transgenic mouse model with the E693Q mutation (APP751) also driven by the Thy1 promoter displayed distinct pathology. Intraneuronal Aβ was detected at 2 months, progressing to intraneuronal lysosomal accumulation of C-terminal fragments (CTFs) and lysosomal abnormalities by 12 months (Gandy et al., 2010; Kaur et al., 2017). At the same time, CAA, loss of cholinergic neurons and GABAergic interneurons, and microglial and astrocyte activation were observed. Notably, this model showed no NFTs or amyloid plaques. These findings highlight the unique pathological timelines and molecular features of APP Dutch models, emphasizing their value in studying Alzheimer’s disease mechanisms and potential therapeutic interventions.

##### 2.13.1.5 V717I (London mutation)

Position 717 in the amyloid precursor protein (APP) is a well-established hotspot for mutations linked to autosomal dominant Alzheimer’s disease (ADAD). Among these, the valine-to-isoleucine substitution (V717I) was one of the first identified mutations, playing a pivotal role in the development of the amyloid cascade hypothesis of AD pathogenesis. While extensively studied in familial cases and used as the foundation for generating widely utilized animal models, detailed neuropathologic data on individuals carrying the V717I mutation remain limited. In this study, we provide a comprehensive clinical and neuropathologic characterization of an APP V717I mutation carrier, shedding new light on the phenotypic variability observed in ADAD cases (Lloyd et al., 2020). The APP(V642I) KI mouse model was engineered by introducing the V717I mutation into exon 17 of the mouse APP gene using homologous recombination and the Cre-loxP system. Biochemical analysis revealed an increased Aβ42/Aβ40 ratio at 29 months, correlating with the onset of memory impairments observed at 27 months. This model provides valuable insights into AD pathogenesis and progression (Kawasumi et al., 2004). These models provide complementary insights into the pathophysiology of AD, highlighting the distinct temporal and biochemical dynamics of amyloid pathology and cognitive decline.

##### 2.13.1.6 SAMP8 mice

SAMP8 (Senescence-Accelerated Mouse Prone 8) mice, a spontaneous AD model, exhibit age-related cognitive decline, oxidative damage, and impaired BBB function. These characteristics make SAMP8 mice particularly suitable for investigating sporadic AD and its progression (Table 6). Overproduction of APP leads to amyloid-β accumulation and plaque formation. Elevated markers of oxidative stress contribute to neuronal damage. Cognitive impairments mimic age-related decline seen in human AD. BBB dysfunction exacerbates disease progression by hindering Aβ clearance.

**TABLE 6:** In vivo-studies using 5 × FAD and SAMP8 Mice.

Classification	Compound/Extract	Source	Experimental Model	Dose; Duration	Efficacy	Mechanism	References
plant	Malva parviflora extract	*Malva parviflora*	5×FAD transgenic mice	50 mg/kg/day; 8 months	Anti-inflammatory, anti-oxidant, anti-dementia	↑CD36	Medrano-Jiménez et al. (2019)
plant	Bazhu Decoction	*Radix Morindae Officinalis* *Fructus Corni* *Pheretima* *Rhizoma Acori, Tatarinowii* *Arisaema cum Bile*	5×FAD transgenic mice, WT	4225, 8450, 16,900 g/kg/day; 12 weeks	Anti-inflammatory, anti-amyloid, anti-oxidant	↓BACE1, PS1-NTF	Peng et al. (2019)
plant	Bushen-Yizhi formula	*Cnidium monnieri L* *Panax ginseng C. A. Mey* *Polygonum multiflorum Thuna* *Paeonia suffruticosa Andr* *Ligustrum lucidum Ait* *Lycium barbarum L*	SAMP8 mice	1.46, -, 5.84 g/kg/day; 4 weeks	Anti-apoptosis, Anti-dementia	↑ChAT, Ach, SIRT1 ↓AChE, PERK, CHOP	Zhang et al. (2017a)
plant	Fuzheng Quxie Decoction	*Ginseng Radix et Rhizoma* *Rhizoma Coptidis* *Rhizoma Ligustici Chuanxiong*	SAMP8 mice	0.7, 3.5 g/mL (extract)	Anti-dementia	↓A*β*	Yang et al. (2019)

5×fad, transgenic mice with 5 familial AD, gene mutation; SAMP8, senescence accelerated mouse-prone 8; M1, macrophage M1.

#### 2.13.2 Therapeutic investigation

Several studies highlight the therapeutic potential of natural compounds in these models. Malva parviflora Extract (5×FAD) reduced microglial pro-inflammatory MI phenotype, promoted phagocytosis via CD36 upregulation, and reduced amyloid plaques. Tested at 50 mg/kg/day for 8 months in 5×FAD mice (Medrano-Jiménez et al., 2019). Bazhu Decoction reduced cognitive and anxiety impairments by inhibiting BACE1 and PS1-NTF protein levels, leading to reduced APP processing and reduced oxidative stress markers. Fuzheng Quxie Decoction reduced Aβ and tau hyperphosphorylation, improved angiogenesis and cerebral blood flow, improving learning and memory in SAMP8 mice. Enhanced angiogenesis and cerebral blood flow via inhibition of HIF1α overactivation. Effective at doses of 0.7–3.5 g/mL or 1.3–2.6 g/kg for up to 12 weeks (Wang F. et al., 2018; Yang et al., 2019). BALB/c, 5×FAD, and SAMP8 mice provide versatile platforms for AD research, enabling precise modeling of genetic, inflammatory, and age-related factors. These models have been instrumental in advancing our understanding of AD pathology and evaluating the efficacy of therapeutic interventions.

#### 2.13.3 ICR mice insights into Alzheimer’s disease research

ICR mice, known for their robust physiology and adaptability, serve as a valuable model in AD research. Their stable genetic background and predictable responses to experimental interventions make them ideal for investigating (Aβ) pathology, tau hyperphosphorylation, neuroinflammation, and oxidative stress. These mice have been used extensively to evaluate the efficacy of natural compounds, uncovering promising therapeutic pathways such as amyloid clearance, inflammation suppression, and cognitive restoration (Table 7).

**TABLE 7:** *In vivo*-Studies using ICR mice.

Classification	Compound/Extract	Source	Experimental model	Dose; duration	Efficacy	Mechanism	References
Plant	Alpinae Oxyphyllae Fructus extract	*Alpinae Oxyphyllae Fructus*	lipopolysaccharide - treated ICR mice	360 mg/kg; 14 days	Anti-oxidative stress, Anti-neuroinflammatory	↓IBA-1, IL-1β, Aβ_1-42_, p-tau	Wang et al. (2018a)
Plant	Annona atemoya leaf extract	*Annona atemoya*	amyloid-β-treated ICR mice	50, 100 mg/kg; 23 days	Anti-amyloid, anti-inflammatory, antioxidant	↓p-EGFR, p-GRK2	Lim et al. (2019a)
Plant	Crataegus pinnatifida fruit	*Crataegus pinnatifida*	β-amyloid protein- treated ICR mice	30, 300 mg/kg	Anti-inflammation, Antioxidant	↓Aβ	Lim et al. (2019a)
Plant	WS-5 Extract	*Curcuma longa, Chaenomeles sinensis, Zingiber officinale*	amyloid- treated ICR mice	250 mg/kg; 14 days	Anti-inflammatory, anti-amyloid, anti-dementia	↓AChE, TNF-*α,* IL-6, A*β*_1-42_	Kim et al. (2019)
Plant	Bojungikgi-Tang	*Hanzung Bojungikgitang Mix Ext. Powder*	Aβ-injected ICR mice	200, 400, 800 mg/kg/day	Anti-amyloid, anti-inflammatory, anti-dementia	↓Aβ	Lim et al. (2018)
Plant	Wu-tou Decoction	*Radix Aconiti* *Herba Ephedrae* *Radix Astragali* *Raidix Paeoniae Alba* *Radix Glycytthizae*	ICR mice	3.15, 6.30, 12.60 g/kg/day; 21 days	Anti-inflammatory, anti-depressant, anti-dementia	↓IL-1β, CCL2, CXCL1	Zhang et al. (2018)

ICR, institute of cancer research mouse; p-EGFR, phosphorylation of epidermal growth factor receptor; p-GRK2, phosphorylated G protein-coupled receptor kinase 2; LPS, lipopolysaccharide; IBA-1, ionized calcium-binding adapter molecule 1; IL-1β, Interleukin 1 beta; Aβ1-42, amyloid-β 1–42; p-tau, phosphorylated tau; AChE, acetylcholinesterase; TNF-α, tumor necrosis factor-α; CCL2, C-C Motif Chemokine Ligand 2; CXCL1, C-X-C Motif Chemokine Ligand 1.

#### 2.13.4 Therapeutic investigations

Wang et al. demonstrated that Alpinae Oxyphyllae Fructus extract significantly alleviated LPS-induced cognitive impairments in ICR mice. Administered at a dose of 360 mg/kg for 14 days, the extract markedly reduced IBA-1, IL-1β, Aβ1-42, and phosphorylated tau (p-tau) levels, highlighting its anti-inflammatory and neuroprotective properties (Wang Y. et al., 2018). Similarly, Lim et al. revealed that Annona atemoya leaf extract suppressed amyloid-β aggregation via inhibition of the p-EGFR and p-GRK2 pathways. ICR mice treated with 50 and 100 mg/kg for 23 days exhibited reduced Aβ accumulation and oxidative stress, showcasing the extract’s dual neuroprotective effects (Lim H.-S. et al., 2019). Further studies by Lim et al. on Crataegus pinnatifida fruit extract showed its ability to protect against memory deficits and glial activation by suppressing Aβ levels. These benefits were observed at doses of 30 and 300 mg/kg in Aβ-treated ICR mice (Lim H. J. et al., 2019). Kim et al. explored WS-5 Extract, a formulation of Curcuma longa, Chaenomeles sinensis, and Zingiber officinale, which demonstrated inhibition of AChE, TNF-α, IL-6, and Aβ1-42. This led to improved cognitive function in ICR mice treated with 250 mg/kg for 14 days (Kim et al., 2019). Bojungikgi-Tang was shown to significantly inhibit Aβ and BACE1 activity, enhancing cognitive performance in Aβ njected ICR mice. Cognitive improvements were evident at doses of 200, 400, and 800 mg/kg/day, as confirmed by passive avoidance and Y-maze tests (Lim et al., 2018). Additionally, Zhang et al. highlighted the neuroprotective efficacy of Wu-tou Decoction, which reduced levels of IL-1β, CCL2, and CXCL1, while mitigating glial cell activation and neuroinflammation. Administered at doses of 3.15, 6.30, and 12.60 g/kg/day over 21 days, the decoction consistently demonstrated beneficial outcomes across varying concentrations. Despite these promising findings, certain limitations persist. For example, studies involving Wu-tou Decoction (Zhang et al., 2018), Crataegus pinnatifida fruit extract (Lim H. J. et al., 2019) and Bojungikgi ang (Lim et al., 2018) provide limited details regarding the duration of treatment, which may hinder reproducibility and comprehensive evaluation of therapeutic potential. Nevertheless, these studies underscore the significant role of natural products in mitigating AD-related pathologies, including neuroinflammation, amyloid aggregation, and cognitive decline. The use of ICR mice as a preclinical model continues to provide critical insights, bridging the gap between basic research and clinical applications in AD therapy.

### 2.14 Kunming mice insights into Alzheimer’s disease research

Kunming mice, closely related to the C57BL strain, are highly valued in AD research for their resilience, adaptability, and high reproductive efficiency. Their genetic stability and robust physiological responses make them an excellent model for investigating AD pathophysiology, including amyloid beta (Aβ) aggregation, neuroinflammation, oxidative stress, and cholinergic dysfunction. These attributes position Kunming mice as a critical preclinical model for evaluating the therapeutic efficacy of natural compounds in mitigating AD-related pathology. In recent years, significant studies have employed Kunming mice to assess the potential of natural products in addressing hallmark features of AD, such as neuroinflammation, Aβ aggregation, and neuronal apoptosis. These findings underscore the therapeutic potential of natural compounds as promising alternatives or complementary treatments for AD. Addressing these gaps is crucial for enhancing reproducibility and understanding the comprehensive therapeutic potential of these compounds (Table 8).

**TABLE 8:** *In vivo*-studies using kunming mice.

Classification	Compound/Extract	Source	Experimental model	Dose; duration	Efficacy	Mechanism	References
Plant	*Lonicera japonica*		LPS-treated Kunming mice	LPS + LJP 30 mg/kg and LPS + LJP 100 mg/kg	Anti-AD	↓ATG5, Beclin 1, Vps34, and LC3 II	Wang et al. (2021a)
Plant	Alpinia oxyphylla—Schisandra chinensis herb pair	*Alpiniaoxyphylla Miq. Fructus* *Schiandra chinensis, Baill Fructus*	Aβ1-42-treated Kunming mice	1200 mg/kg	Anti-inflammatory, anti-apoptotic	↓NF-κB	Qi et al. (2019)
Plant	Schisandra chinensis Extract	*Schisandra chinensis*	Kunming mice	10 mg/kg; 14 days	Antioxidant, anti-cholinesterase, Anti-AD	↓AChE	Song et al. (2020)
Plant	Ethyl Acetate Extract Components of Bushen-Yizhi Formula	*common Cnidium fruit, tree peony bark, ginseng root, Radix Polygoni Multiflori Preparata, barbary wolfberry fruit and Fructus Ligustri Lucidi*	Kunming mice	1.46, 2.92, 5.84 mg/kg; 17 days	Anti-cholinesterase, anti-apoptotic, antioxidant	↓AChE, ChAT	Zhang et al. (2017b)
Plant	Kai-Xin-San	*Panax ginseng C. A. Mey* *Polygala tenuifolia Willd* *Acorus tatarinowii* *Poria*	Kunming mice	1.4, 2.8 g/kg; 14 days	Anti-amyloid, anti-neuroinflammatory	↓BACE1	Luo et al. (2020)

Aβ1-42, amyloid-β 1–42; NF-κB, nuclear factor kappa-light-chain-enhancer of activated B cells; AChE, acetylcholinesterase; SCOP, scopolamine; ChAT, choline acetyltransferase; Aβ, amyloid-β; BACE1, Beta-Secretase 1.

#### 2.14.1 Therapeutic investigations

Several groundbreaking investigations have revealed the neuroprotective effects of natural compounds using Kunming mice. These studies emphasize the ability of these compounds to address various aspects of AD pathology. *Lonicera japonica* (*L. japonica*), a renowned traditional Chinese herbal medicine, is widely recognized for its anti-inflammatory properties. However, its potential role in neuroprotection remains poorly understood. Polysaccharides, identified as the primary bioactive components of *L. japonica*, have garnered attention for their therapeutic potential. Recent research has investigated the effects of *L. japonica* polysaccharides (LJP) on cognitive impairment induced by lipopolysaccharide (LPS) and explored the underlying molecular mechanisms. This study sheds light on LJP’s potential as a neuroprotective agent, offering promising insights into its role in mitigating cognitive deficits associated with neuroinflammation (Wang J. et al., 2021). Qi et al. demonstrated the efficacy of *Alpinia oxyphylla–Schisandra chinensis* herb pair in reducing apoptosis and inflammation in Aβ1-42-treated Kunming mice. Administered at a dose of 1200 mg/kg, the treatment significantly downregulated NF-κB levels, a key driver of neuroinflammation, showcasing strong anti-apoptotic and anti-inflammatory effects (Qi et al., 2019). Song et al. reported that *S. chinensis* extract (10 mg/kg for 14 days) mitigated scopolamine-induced cholinergic deficits and oxidative stress. This extract effectively inhibited AChE activity, restoring cognitive function and highlighting its neuroprotective potential (Song et al., 2020). Zhang et al. confirmed that Ethyl Acetate Extract Components of Bushen-Yizhi Formula improved learning and memory impairments while enhancing cholinergic system function. The treatment, administered at doses of 1.46, 2.92, and 5.84 mg/kg over 17 days, reduced AChE levels and increased ChAT levels, reversing scopolamine-induced cognitive deficits (Zhang et al., 2017a). Luo et al. revealed that Kai-Xin-San effectively inhibited Aβ generation and aggregation by reducing BACE1 levels in scopolamine-treated Kunming mice. The treatment, administered at doses of 1.4 and 2.8 g/kg for 14 days, demonstrated significant efficacy in mitigating AD-related amyloid pathology (Luo et al., 2020). While these studies demonstrate compelling evidence of natural compounds’ efficacy, limitations remain, including insufficient clarity on dosing duration in certain investigations, such as those by Qi et al. (2019).

### 2.15 C57BL/6 mice insights into Alzheimer’s disease research

C57BL/6 mice are the most extensively used models in AD research, owing to their exceptional genetic stability, robust breeding capabilities, and adaptability to laboratory conditions. These mice have proven invaluable in investigating the molecular mechanisms underlying AD pathology and assessing the efficacy of therapeutic interventions, particularly natural products. Their genetic background makes them highly suited for exploring core aspects of AD pathology, such as amyloid-beta (Aβ) aggregation, tau hyperphosphorylation, neuroinflammation, synaptic dysfunction, and cognitive decline. C57BL/6 mice have served as the foundation for numerous preclinical studies, enabling researchers to gain insights into the complex interplay of pathological processes in AD. Seven pivotal studies utilizing this model have demonstrated the multifaceted neuroprotective effects of natural products, as detailed below (Table 9).

**TABLE 9:** *In vivo*-studies using C57BL/6 mice.

Compound/Extract	Source	Experimental model	Dose; duration	Efficacy	Mechanism	References
Bu-Shen-Yi-Sui Capsule	*Rehmanniae* *Radix* *Rehmanniae Radix Praeparata* *Polygoni Multiflori Radix* *Rhei Radix et Rhizoma, Leonuri Herba* *Fritillariae Thunbergii Bulbus, Hirudo* *Scorpio, Gastrodiae Rhizoma, Forsythiae Fructus*	C57BL/6 mice	3.02 g/kg; 40 days	Anti-inflammation	↑MBP	Zhao et al. (2020)
Huang-Lian-Jie-Du Decoction	*Rhizoma Coptidis* *Cortex Phellodendri* *Fructus Gardeniae* *Salvia miltiorrhiza* *Curcuma longa L., Acorus tatarinowii*	C57BL/6 mice	3.5, 7 g/kg; 3 weeks	Cognitive improvement, synaptic health	↑NR1, NR2A, NR2B	Liu et al. (2019)
Kamikihito	*14 medicinal herbs including* *Ginseng Radix* *Astragali Radix*	C57BL/6J Mice	0, 1, 2 g/kg; 7 days	Reduced anxiety, improved hedonic behavior		Oizumi et al. (2020)
Soshiho-tang	*Bupleuri Radix, Scutel- lariae Radix, Ginseng Radix, Pinelliae Tuber, Glycyrrhizae Radix et Rhizoma, Zingiberis Rhizoma Crudus* *Zizyphi Fructus*	amyloid-beta-treated C57BL6 mice	500–2000 mg/kg/day; 20 days	Neuroprotective, Anti-inflammatory effects	↓Aβ, AChE	Sohn et al. (2021)
Tongqiaohuoxue	Salvia miltiorrhiza, Angelica sinensis, Ligusticum chuanxiong	C57BL/6J and ApoE-deficient mice	100 mg/kg; 8 weeks	Reduced plaques, improved lipid metabolism	↓AchE	Ha et al. (2020)
Xueshuantong	Panax notoginseng, Radix Astragali, Ligusticum chuanxiong	APPswe/PSEN1dE9 mice (C57BL/6)	100 mg/kg; 30 days	Improved learning and blood flow	↓Aβ	Huang et al. (2018)
Yokukansan	Atractylodes lancea, Poria cocos, Ziziphus jujuba	C57BL6/J mice 5×FAD mice	Prepared in wet food pellets	Reduced astrogliosis, improved behavior	↓Astrogliosis	Kaushik et al. (2018)

C57BL/6 mice, common inbred strain of laboratory mouse; MBP, myelin basic protein; APPswe/PSEN1dE9 mice, Swedish mutation of APP; Aβ, amyloid-β; C57BL/6J mice, common inbred strain of laboratory mouse; AChE, acetylcholinesterase; NR1, N-Methyl-D-Aspartate receptor 1; NR2A N-methyl-D-aspartate receptor 2A; NR2B, N-methyl-D-aspartate receptor 2B HFHC, high-fat and high-cholesterol; 5×FAD, mice, 5 familial AD, mutations mice.

#### 2.15.1 Therapeutic investigations

A series of innovative studies highlight the potential of natural compounds in addressing AD pathology using C57BL/6 mice. Zhao et al. demonstrated that Bu-Shen-Yi-Sui Capsule effectively inhibited inflammatory infiltration in brain tissue and preserved the ultrastructural integrity of myelin. These effects were mediated by the upregulation of myelin basic protein (MBP) in C57BL/6 mice treated with 3.02 g/kg of the capsule for 40 days, underscoring its neuroprotective potential (Zhao et al., 2020). Liu et al. reported that Huang-Lian-Jie-Du Decoction significantly improved memory deficits by upregulating NR1, NR2A, and NR2B levels. The treatment, administered at doses of 3.5–7 g/kg for 3 weeks, effectively attenuated cognitive decline, highlighting its role in synaptic plasticity and neuronal health (Liu et al., 2019). Oizumi et al. demonstrated that *Kamikihito* reduced anxiety levels and enhanced consummatory hedonic responses in C57BL/6J mice. These dose-dependent effects were observed at 0, 1, and 2 g/kg over 7 days, showcasing its potential to address AD-associated anxiety and emotional dysfunction (Oizumi et al., 2020). Sohn et al. showed that Soshiho-tang exhibited both neuroprotective and anti-inflammatory effects by downregulating Aβ and AChE levels. Administered at doses of 500–2000 mg/kg/day for 20 days, the treatment provided strong evidence of its neuroprotective efficacy (Sohn et al., 2021). Ha et al. found that *Tongqiaohuoxue* reduced atherogenic plaque formation and lipid deposition induced by a high-fat, high-cholesterol diet. Furthermore, it attenuated Aβ plaque formation by suppressing AChE activity in ApoE-deficient and WT C57BL/6J mice treated with 100 mg/kg for 8 weeks (Ha et al., 2020). Huang et al. reported that *Xueshuantong* enhanced spatial and motor learning and memory functions by improving cerebral blood flow and reducing amyloid plaque density and size. These effects were observed in APPswe/PSEN1dE9 mice with a C57BL/6 background treated with 100 mg/kg for 30 days, demonstrating its potential role in vascular health and AD symptom management (Kaushik et al., 2018). Kaushik et al. confirmed that *Yokukansan* attenuated behavioral impairments and reduced astrogliosis in C57BL/6J and 5×FAD mice. However, the study lacked essential methodological details, including dosage and treatment duration, limiting the reproducibility and interpretability of the findings (Kaushik et al., 2018).

### 2.16 Sprague dawley rat insights into Alzheimer’s disease research

Sprague Dawley rats, distinguished by their white fur and red eyes, are a cornerstone in AD research. Their rapid growth, high fertility, and low incidence of tumors, coupled with their sensitivity to hormonal changes, make them ideal for studying neurological, cardiovascular, and endocrinal disorders. In the realm of AD research, these rats have proven indispensable for unraveling molecular mechanisms of disease progression and evaluating the therapeutic potential of natural compounds. Over the years, studies using Sprague Dawley rats have elucidated therapeutic pathways targeting neuroinflammation, amyloid-beta (Aβ) aggregation, tau hyperphosphorylation, oxidative stress, and synaptic plasticity. This model has provided a robust platform for testing natural products and their ability to mitigate AD pathology (Table 10).

**TABLE 10:** In vivo-studies using Sprague Dawley rat.

Classification	Compound/Extract	Source	Experimental model	Dose; duration	Efficacy	Mechanism	References
Plant	Rhodiola crenulata extract (RCE)	*Rhodiola crenulata*	STZ injected SpragueDawley rat	1.5–6.0 g/kg; 21 days	Neuroprotective, anti-apoptotic	↑ ATP, CcO ↓ Apoptotic neurons	Wang et al. (2017a)
Plant	Rhodiola crenulata extract	*Rhodiola crenulata*	Aβ_1-42_ injected rat	0.56–2.24 g/kg; 28 days	Anti-Alzheimer, antioxidant	↑ACh, ↓AchE, ↓p-tau	Zhang et al. (2019b)
Plant	Huannao Yicong Decoction (HYD)	*Huannao* *Yicong*	A*β*_1–42_ injected rat	3.78–18.90 mg/kg; 12 weeks	Anti-Alzheimer, anti-tau	↓Aβ, ↓TTBK1, ↓CDK-5, ↓GSK-3β	Cao et al. (2016)
Plant	Kai-Xin-San Decoction	*Polygala tenuifolia, Panax ginseng, Poria cocos*	Aβ_25-35_ injected rat	2–10 g/kg; 21 days	Anti-inflammatory, anti-apoptotic	↑ Bax, c-caspase-3 ↓ IL-1β, TNF-α, p-Tau	Guo et al. (2019)
Plant	Sanweidoukou Decoction	*Sanweidoukou*	Aβ_25-35_ injected SpragueDawley rat	0.5–2 g/kg; 28 days	Anti-inflammation Anti Aβ	↓ IL-6, COX-2, IL-1β	An et al. (2021)
Plant	Xiaoyao Pills	*Xiaoyao*	OB-induced rats	0.93–1.86 g/kg; 30 days	Antidepressant, anti-inflammatory	↓IL-6, ↓IL-1β, ↓MDA	Ji et al. (2021)
Plant	Xiaoyaosan Decoction	*Xiaoyaosan*	SpragueDawley rat	0.0197 g/100 g; 21 days	Antidepressant	↑ PREG ↓ PROG, ALLO	Guo et al. (2017)
Plant	Xiaoyaosan Decoction	*Xiaoyaosan*	CUMS rats	2.224 g/kg; 21 day	Antidepressant	↑ MAP2, NR2B, PI3K ↓ Glutamate	Zhou et al. (2021a)
Plant	Xuefu Zhuyu Decoction	*Xuefu zhuyu*	CCI-induced rats	9–18 g/kg; 21 days	Cognitive recovery, neuroregeneration	↑ NMDAR1	Zhou et al. (2017)
Plant	Zuoguiwan decoction	*Zuoguiwan*	STZ-injected rats	9.45 g/kg; 7 days	Anti-inflammation, Antioxidant	↓ IL-1β, TNF-α, IL-6	Liu et al. (2021)

PREG, pregnenolone; PROG, progesterone; ALLO, alloprognanolone; ATP, adenosine triphosphate; CcO, cytochrome c oxidase; IL-6, Interleukin 6; COX-2, cyclooxygenase-2; IL-1β, Interleukin 1 beta; Bax, Bcl-2-associated X protein; c-caspase-3, cleaved-caspase-3; TNF-α, tumor necrosis factor alpha; p-tau, tau protein; Aβ, amyloid beta; TTBK1, Tua Tubulin Kinase 1; CDK-5, Cyclin dependent Kinase 5; GSK-3*β*, glycogen synthase kinase 3*β*; Ach, acetylcholine; ChAT, choline acetyl transferase; AchE, acetylcholinesterase; MDA, malondialdehyde; NO, nitric oxide; MAP2, microtubule associated protein 2; NR2B, N-methyl-D-aspartate receptor 2B subunit; PI3K, phosphoinositide 3-kinase; NMDAR1, N-methyl-D-aspartate receptor 1.

#### 2.16.1 Therapeutic investigations

Wang et al. demonstrated the neuroprotective potential of *Rhodiola crenulata* Extract (RCE) in 90 female Sprague Dawley rats across five experimental groups. RCE significantly increased ATP and cytochrome c oxidase levels while reducing mitochondrial injury and neuronal apoptosis. These findings underscore its efficacy in combating AD-related neurodegeneration (Wang X. et al., 2017). Zhang et al. revealed that RCE improved ACh levels, enhanced ChAT activity, and reduced malondialdehyde (MDA) and phosphorylated tau (p-tau) levels. The treatment also induced p-GSK3β expression, demonstrating its multifaceted approach to alleviating AD pathology (Zhang X. et al., 2019). Tested in 72 Sprague Dawley rats, *Huannao Yicong* Decoction (HYD) demonstrated superior efficacy compared to donepezil by reducing Aβ levels, tau phosphorylation, and expression of tau protein kinase markers (TTBK1, CDK-5, GSK-3β) in the hippocampal CA1 region. This highlights its potential to halt neurodegeneration and enhance cognitive function (Cao et al., 2016). Guo et al. found that Kai-Xin-San (KXS) ameliorated AD symptoms by reducing tau hyperphosphorylation, AChE levels, TNF-α, IL-1β, and ROS levels, while inhibiting caspase-3 cleavage. These findings emphasize KXS’s potential in regulating neuroinflammation and preventing neuronal apoptosis (Guo et al., 2019). Sanweidoukou Decoction (DK-3) displayed significant neuroprotective effects in both *in vitro* and *in vivo* models of Aβ-induced AD pathology. *In vivo*, DK-3 treatment reduced tau phosphorylation (Thr181, Thr205, Ser396) and inflammation markers such as IL-6, CoX-2, and IL-1β, alleviating neuronal damage (An et al., 2021). Xiaoyao Pills (XYW) alleviated depression-like behaviors in olfactory bulbectomy (OB)-induced AD models. XYW effectively reduced superoxide dismutase activity, glutathione levels, malondialdehyde, nitric oxide, and pro-inflammatory cytokines (IL-6, IL-1β), supporting its traditional use in treating anxiety and mood disorders (Ji et al., 2021). Xiaoyaosan (XYS) demonstrated therapeutic efficacy in chronic unpredictable mild stress (CUMS) models. XYS reduced PREG, PROG, and ALLO levels (Guo et al., 2017). Improving hippocampal and amygdala function and ameliorating depressive-like behaviors associated with cognitive decline (Zhou X. M. et al., 2021). Xuefu Zhuyu Decoction (XFZYD) enhanced cognitive recovery in controlled cortical impact (CCI)-induced trauma models. Administered at 9–18 g/kg/day, XFZYD upregulated NMADR1, CaMKII, and GAP-43, effectively improving neurological deficits and cognitive impairments (Zhou et al., 2017). Liu et al. reported that Zuoguiwan (ZGW) improved streptozotocin (STZ)-induced cognitive impairments. ZGW reduced escape latency, increased time spent in the target quadrant, and lowered pro-inflammatory cytokines (IL-6, IL-1β, TNF-α), implicating estrogen receptor β (ERβ) in its therapeutic mechanisms (Liu et al., 2021).

### 2.17 Wistar rats insights into Alzheimer’s disease research

Wistar rats, an outbred albino strain distinguished by their white fur, red eyes, and characteristic physical features, are a cornerstone model in neuroscience and pharmacology. Known for their high activity levels, tumor resistance, and adaptability, these rats are invaluable for studying AD. Their robust physiological attributes make them ideal for exploring disease mechanisms and evaluating therapeutic interventions. In AD research, Wistar rats have been instrumental in understanding critical aspects of neuroinflammation, tau hyperphosphorylation, amyloid-beta (Aβ) aggregation, oxidative stress, and cholinergic dysfunction. Several studies have focused on natural product-based therapies, revealing promising mechanisms of action such as antioxidant activity, neuroprotection, and inflammation modulation. These findings contribute to developing novel therapeutic strategies targeting AD pathology (Table 11).

**TABLE 11:** *In vivo* studies using Wistar Rat.

Classification	Compound/Extract	Source	Experimental Model	Dose; duration	Efficacy	Mechanism	References
Plant	*Capparis spinose* extract	*Capparis spinosa*	Aβ injected rats	20 mg/kg/BW; 6 weeks	Anti-inflammatory	↓APP ↓BACE-1 ↓PSEN-1/2	(Mohebali et al.)
Plant	Methanolic Extract (*Caesalpinia crista*)	*Caesalpinia crista*	AlCl_3_ -treated rats	100–400 mg/kg; 60 days	Anti-inflammatory, antioxidant	↓AChE ↓IL-1β ↓TNF-α	Ravi et al. (2018)
Plant	*Harrisonia abyssinica* extract	*Harrisonia abyssinica*	AlCl_3_ -treated rats	100–200 mg/kg; 3 weeks	Neurotransmitter normalization, anti-oxidant	↑Dopamine ↓MDA	Anwar et al. (2021)
Plant	*Rosa canina* *Tanacetum vulgare* *Urtica dioica*	Combination Extract	Wistar rat	20 mg/kg; 21 days	Anti-inflammatory, antioxidant	↓ TNF-α	Daneshmand et al. (2016)
Plant	Hachimijiogan (HJG) Decoction	*Hachimijiogan*	Aβ injected rats	100–1000 mg/kg; 7 days	Anti-amyloidogenic, neuroprotective	↑ CREB	Kubota et al. (2017)
Plant	Shenzhi Jiannao decoction	*Shenzhi jiannao*	Wistar rat	1.89–7.56 g/kg; 7 days	Anti-inflammation, Anti-oxidant, Anti-apoptosis activity	↓ Ca^2+^ ROS	Tian et al. (2021)
Plant	Yizhi Qinxin Formula (YQF)	*Yizhi Qingxin*	Wistar rat	0.3–0.6 mg/kg; 8 weeks	Anti-inflammatory, neurotrophic effects	↑ACh ↓TNF-α ↓IL-6	Ma et al. (2020)

MDA, malondialdehyde; IL-1β, Interleukin 1 beta; CREB, cAMP, response element binding protein; ROS, reactive oxygen species; TNF-α, tumor necrosis factor alpha; AChE, acetylcholinesterase; IL-6, Interleukin 6; Ach, acetylcholine; IL-10, Interleukin 10; IL-2, Interleukin 2.

#### 2.17.1 Therapeutic investigations

Mohebali et al. evaluated the antioxidant activity of *Capparis spinosa* using DPPH and FRAP assays. In Aβ-induced Wistar rats, RT-PCR analysis revealed downregulation of APP, BACE-1, PSEN-1, and PSEN-2 gene expression in the Aβ+/CS+ (*C. spinosa*-treated) group. However, the limited sample size (n = 3 per group) reduces the reliability of these findings (Mohebali et al., 2018). Methanolic extracts of *C. crista* (MECC) demonstrated neuroprotective effects by improving escape latency times and alleviating oxidative stress markers (catalase, GSH, GST) in AlCl_3_-treated rats. MECC also suppressed AChE activity and reduced pro-inflammatory cytokines (TNF-α, IL-1β, IL-6) in the frontal cortex and hippocampus, indicating its therapeutic efficacy (Ravi et al., 2018). *Harrisonia abyssinica* leaf extract restored hippocampal AChE, ERK, glutamate, and MDA levels in AlCl_3_-treated rats. The treatment normalized neurotransmitter levels (norepinephrine, dopamine, serotonin), highlighting its potential in ameliorating AD-like symptoms (Anwar et al., 2021). Daneshmand et al. demonstrated improved spatial learning and reduced Psen1 gene expression in the hippocampus of STZ-induced sporadic AD (SAD) rats. However, the study’s robustness was limited by small group sizes, incomplete statistical data on MapK and TNF-α, and contradictory findings regarding gene expression differences (Daneshmand et al., 2016). Kubota et al. investigated Hachimijiogan (HJG), revealing its ability to induce neurite outgrowth in PC12 cells by acting as a nerve growth factor (NGF). *In vivo*, HJG shortened swim times in the Morris Water Maze and increased phosphorylated CREB levels in Aβ-injected Wistar rats, demonstrating its neuroprotective potential (Kubota et al., 2017). Tian et al. reported that Shenzhi Jiannao Formula (SZJNF) significantly reduced intracellular calcium, ROS, and superoxide levels in glutamate-treated PC12 cells. In vascular dementia (VD) rats, SZJNF improved neuronal morphology, reduced synaptic dysfunction, and attenuated oxidative stress and apoptosis via calcium signaling pathways, highlighting its potential in treating AD-related neurodegeneration (Tian et al., 2021). Ma et al. demonstrated that Yizhi Qinxin Formula (YQF) improved cognitive function in the Morris Water Maze test. YQF increased normal neuron counts, reduced inflammatory cytokines (TNF-α, IL-2, IL-6), and elevated BDNF, TrkB, and NGF mRNA expression. Additionally, YQF enhanced ACh levels and reduced β-AP expression, underscoring its role in alleviating AD-like symptoms (Ma et al., 2020).

### 2.18 Lewis rat insights into Alzheimer’s disease research

The Lewis rat, an albino strain widely used in immunological and transplantation studies, holds significant value in research focusing on inflammation-related pathologies. Known for its susceptibility to immune-mediated diseases and distinct immune response profile, the Lewis rat serves as a specialized model for investigating neuroinflammation and related neurodegenerative processes. Although its use in AD research is relatively limited, its unique immunological traits offer valuable insights into inflammatory mechanisms underlying AD pathology (Table 12).

**TABLE 12:** *In vivo* Lewis Rat.

Classification	Compound/Extract	Source	Experimental Model	Dose; Duration	Efficacy	Mechanism	References
Plant	Yisui Tongjing decoction	Yisui tongjing	Experimental autoimmune neuritis Lewis’s rat	1, 1.5, 2.0 mL/d; 4 weeks	Anti-inflammation		Zhang et al. (2016a)

#### 2.18.1 Therapeutic investigations

Zhang et al. explored the therapeutic potential of Yisui Tongjing (YSTJ) in treating Guillain–Barré Syndrome (GBS), a neuroinflammatory condition with overlapping pathological features observed in AD. The study demonstrated that YSTJ administration significantly improved clinical symptoms, enhanced sciatic nerve conduction velocity (NCV), and reduced sciatic nerve demyelination. These beneficial effects were observed in a dose-dependent manner, emphasizing YSTJ’s ability to modulate inflammatory pathways and promote nerve repair (Zhang Y. et al., 2016). While this investigation was not specifically focused on AD, the observed anti-inflammatory and neuroprotective effects suggest that YSTJ may hold therapeutic potential for Alzheimer’s disease. Given the critical role of neuroinflammation in AD pathology, further research using Lewis’s rat models could uncover YSTJ’s efficacy in modulating microglial activity, reducing pro-inflammatory cytokines, and preserving neuronal integrity. This initial evidence highlights a promising avenue for extending the application of YSTJ into AD research, addressing key pathological mechanisms such as neuroinflammation and synaptic dysfunction.

### 2.19 *Drosophila melanogaster* insights into Alzheimer’s disease research

*Drosophila melanogaster*, commonly known as the fruit fly, has emerged as a highly versatile and efficient model organism in AD research. Its genetic simplicity, short life cycle, ease of genetic manipulation, and high reproductive capacity provide researchers with a cost-effective platform for investigating complex neurodegenerative mechanisms. The fly’s nervous system shares significant functional and structural similarities with mammals, making it an ideal model for studying the molecular pathways underlying AD pathology. Furthermore, transgenic *Drosophila* models expressing human AD-associated genes, such as amyloid-beta (Aβ) and tau, have enabled precise exploration of disease progression and therapeutic interventions (Table 13).

**TABLE 13:** *In vivo* studies using *Drosophila melanogaster* fly.

Classification	Compound/Extract	Source	Experimental Model	Dose; Duration	Efficacy	Mechanism	References
Plant	Polyphenolic extract	*Arabidopsis thaliana*	Aβ_1-42_ expressing *Drosophila melanogaster* flies	40 μL/mL; entire developmental period 12 days	Anti-inflammation, Anti-oxidant	↑IL-4, IL-10, IL-13 ↓IL-6, IL-1β, TNF-α	Mattioli et al. (2019)

IL-4, Interleukin 4; IL-10, Interleukin 10; IL-13, Interleukin-13; IL-6, Interleukin-6; IL-1β, Interleukin 1 beta; TNF-α, tumor necrosis factor alpha.

#### 2.19.1 Therapeutic investigations

A study by Zhang et al. investigated the therapeutic potential of polyphenolic extracts derived from *Arabidopsis thaliana* in an AD *Drosophila* model expressing Aβ1–42. The extract demonstrated significant anti-inflammatory and neuroprotective effects by modulating heme oxygenase-1 (HO-1) mRNA expression and enhancing NAD(P)H: quinone oxidoreductase 1 (NQO1) activity. These molecular changes contributed to the suppression of oxidative stress and inflammation, two critical drivers of AD pathology. Additionally, the treated flies exhibited notable improvements in climbing ability, a behavioral marker of motor function in *Drosophila* models, indicating restored neuronal health and functional recovery (Mattioli et al., 2019). These findings underscore the effectiveness of *D. melanogaster* as a robust preclinical model for screening anti-AD compounds. The observed neuroprotective effects of *A. thaliana* polyphenolic extract highlight its potential for mitigating AD-like pathologies through inflammation suppression and oxidative stress reduction. Future studies leveraging the genetic tractability of *Drosophila* could further dissect the molecular pathways influenced by such natural compounds, accelerating the discovery of novel AD therapeutics.

### 2.20 *Danio rerio* (zebrafish) models insights into Alzheimer’s disease research

The zebrafish (*Danio rerio*) has emerged as an indispensable model organism for studying neurodegenerative diseases, including AD (Shenoy et al., 2022). Its unique combination of genetic similarity to humans, transparent embryos, and neuroanatomical parallels makes it a powerful platform for investigating AD mechanisms and testing therapeutic compounds. The zebrafish genome contains orthologs for approximately 70% of human disease-related genes, including those implicated in AD, such as *APP*, *PSEN1*, and *PSEN2*. Additionally, their small size, rapid reproduction cycle, and compatibility with high-throughput screening techniques provide unmatched efficiency in large-scale drug discovery studies (Koehler D, 2018). Behavioral assays, including memory-based tasks and spatial navigation tests, are easily adapted to zebrafish models, enabling detailed assessments of cognitive function and neurobehavioral changes. These attributes, coupled with cost-effectiveness and ethical advantages, have cemented zebrafish as a pivotal tool in AD research. This review compiles insights from ten key studies investigating the therapeutic effects of natural compounds using zebrafish models (Table 14).

**TABLE 14:** *In vivo Danio rerio* (zebrafish).

Classification	Compound/Extract	Source	Experimental Model	Dose; Duration	Efficacy	Mechanism	References
Plant	Agathisflavone	*Schinus polygamus*	Adult zebrafish	1–5 μg/L 9 days	Antioxidant action		Dumitru G et al. (2019)
Plant	Andrographolide	*Andrographis paniculata*	Zebrafish Embryos and Larvae (AB strain)	10–40 μM 24 h	Anti-inflammation, Anti-oxidant	↑ CypA/MMP-9	Zhou X et al. (2024)
Plant	Essential oil	*Glaucosciadium cordifolium*	Adult zebrafish	25–150 μL/L 17 days	Antioxidant, Anti-AchE, Antidepressant		Boiangiu et al. (2023)
Plant	Gac fruit	*Momordica cochinchinensis*	Adult zebrafish	200 mg/kg	Anti-memory impairment activity, Antioxidant, Anti-inflammation, Anti-apoptosis		Singsai K and Chaikhumwang (2024)
Plant	Genistein, quercetin, abietic acid, β-sitosterol	*Cistanche tubulosa*	Adult zebrafish (AB strain)	50–200 μM 05 days	Antioxidant, Anti-inflammation		Li YQ et al. (2020)
Plant	Leaf extract	*Lantana camara Linn*	Adult zebrafish (AB strain)	10, 30, 100 mg/kg 07 days	Antioxidant, Anti-inflammation		Amoah et al. (2023)
Plant	Leaf extract	*Streblus asper*	Adult zebrafish	200–800 mg/kg 07 days	Antioxidant, Anti-inflammation, Anti-Parkinson activities		Singsai et al. (2021)
Plant	Leaf extract	*Guiera senegalensis*	Adult zebrafish	1–8 μg/L 19 days	Antimicrobial activity, Anti-inflammation		Dzoyem et al. (2012)
Plant	Leaf extract	*Lycopodium selago L*	Adult zebrafish	0.5–3 mg/L 14 days	Antioxidant		Valu et al. (2021)
Plant	Root extract	*Convolvulus pluricaulis*	Zebrafish larvae and adults	0.38 mg/mL 7 days	Anti-AchE		Karunakaran et al. (2022)

AChE, acetylcholinesterase; CypA, Cyclophilin A; MPP-9, Matrix metalloproteinase 9.

#### 2.20.1 Therapeutic investigations

**FIGURE 4:**
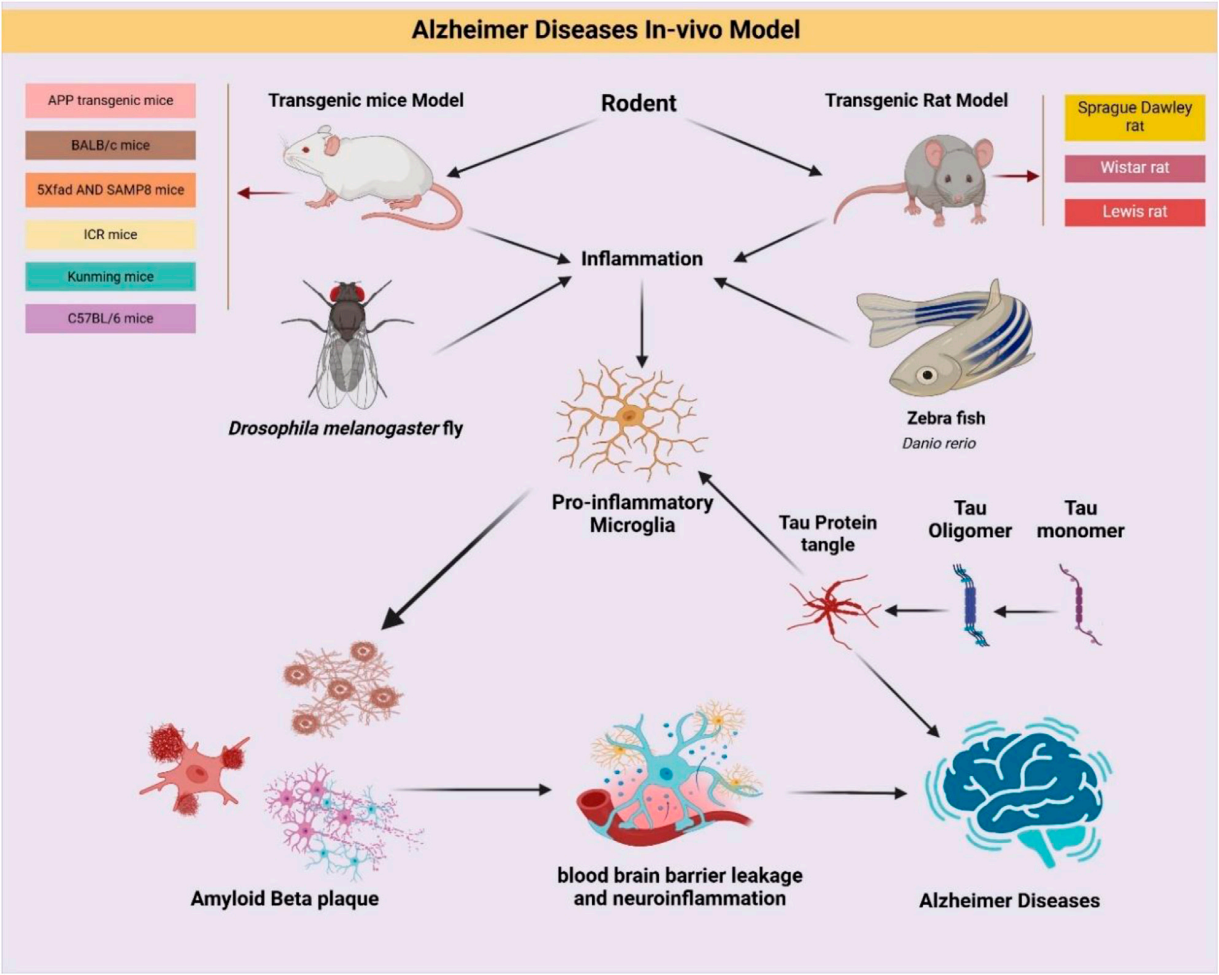
The following picture demonstrates the main characteristics of animal models related to Alzheimer’s disease (AD): Transgenic rats and mice are used as vertebrate models, whereas *Drosophila melanogaster* and zebrafish are used as invertebrate models. Interestingly, AD only develops spontaneously in canine models and non-human primates (NHPs).

Dumitru et al. highlighted the neuroprotective potential of agathisflavone, a flavonoid that reduced anxiety and modulated AChE activity in adult zebrafish. The findings suggest its potential to counteract cognitive deficits associated with AD (Dumitru G et al., 2019). Zhou et al. demonstrated the efficacy of andrographolide, a diterpene lactone, in protecting the blood-brain barrier by inhibiting the CypA-NF-κB-MMP-9 pathway, highlighting its dual anti-inflammatory and neuroprotective effects (Zhou X et al., 2024). Boiangiu et al. (2023) revealed that the essential oil from Glaucosciadium cordifolium reduced oxidative stress, decreased AChE activity, and restored cholinergic dysfunction, showcasing its neuroprotective properties (Boiangiu et al., 2023). Singsai et al. reported that Momordica cochinchinensis fruit extract improved spatial and fear-based memory, suggesting a role in cognitive resilience and AD prevention (Singsai K and Chaikhumwang, 2024). Li et al. evaluated compounds such as genistein, quercetin, abietic acid, and β-sitosterol derived from *Cistanche tubulosa*. These natural compounds modulated tp53 mRNA expression, demonstrating their efficacy in key neuroprotective pathways (Li YQ et al., 2020). Amoah et al. (2023) demonstrated the therapeutic potential of *Lantana camara* Linn. leaf extract, which effectively protected zebrafish from scopolamine-induced memory impairments, highlighting its cognitive-enhancing effects (Amoah et al., 2023). Singsai et al. further reported that *Streblus asper* leaf extract alleviated memory deficits and promoted neuroprotection in zebrafish models (Singsai et al., 2021). Damo et al. investigated the effects of Guiera senegalensis leaf extract, showing improvements in AChE activity and reductions in oxidative stress after 19 days of treatment (Dzoyem et al., 2012). Valu et al. (2021) demonstrated that *Lycopodium selago L*. leaf extract exhibited potent antioxidant activity and significantly mitigated induced memory deficits in zebrafish, reinforcing its therapeutic value (Valu et al., 2021). Karunakaran et al. revealed that *Convolvulus pluricaulis* root extract effectively inhibited AChE activity in both adult and larval zebrafish, suggesting its utility in addressing cholinergic dysfunction in AD (Karunakaran et al., 2022). These studies collectively underscore the versatility and effectiveness of zebrafish in modeling Alzheimer’s disease. Natural compounds evaluated in these models have demonstrated promising results across key pathological pathways, including, reduction of oxidative stress, inhibition of AChE, modulation of inflammatory pathways, preservation of blood-brain barrier integrity, enhancement of spatial and memory-related behaviors. Zebrafish provide a robust platform for preclinical screening and validation of anti-AD therapeutics. Their unique attributes enable researchers to bridge the gap between cellular models and mammalian systems, accelerating the discovery of effective interventions for AD. Numerous animal models have been created to investigate AD, including vertebrate transgenic rodents, invertebrates such as zebrafish and *Drosophila melanogaster*, as well as naturally occurring models in NHPs and dogs (Figure 4).

### 2.21 Clinical trial insights into Alzheimer’s disease research

Clinical trials form the cornerstone of translational research, bridging the gap between preclinical findings and practical therapeutic applications. They offer critical, evidence-based insights into the safety, efficacy, and mechanisms of natural products in addressing AD. To date, 13 significant clinical studies have explored the therapeutic potential of natural compounds in alleviating cognitive decline, managing neuropsychiatric symptoms, and enhancing the quality of life for individuals with AD and age-related cognitive impairments (Table 15). These trials collectively underscore the multifaceted role of natural products in targeting hallmark pathological mechanisms, such as amyloid-beta (Aβ) plaque aggregation, oxidative stress, neuroinflammation, and neurotransmitter dysregulation.

**TABLE 15:** Human studies.

Classification	Compound/Extract	Source	Patients	Dose; duration	Efficacy	Mechanism	References
Plant	Diosgenin-rich yam extract	*Dioscorea batatas*	28	50 mg/day; 12 weeks	Anti-Alzheimer activity Anti-hyperlipidemia	↑ Synapses density	Tohda et al. (2017)
Plant	Polyphenolic extract	*Mentha spicata* L	90	600, 900 mg/day; 90 days	Antioxidant Anti-inflammation	↓ Aβ aggregation	Herrlinger et al. (2018)
Plant	PM-EE	*Salicornia europaea* L	63	600 mg/day; 12 weeks	Anti-amnesic, Anti-depressant, Antioxidant	controls pro-apoptotic processes to prevent neuronal apoptosis	Lee et al. (2020)
Plant	Powder, extract	Wild blueberry	122	500, 1000 mg/day; 100 mg; 3, 6 months	Antioxidant, Antidepressant	↓ Oxidative damage to mitochondria	Whyte et al. (2018)
Plant	extract	*Withania somnifera* (L.) Dunal	50	600 mg/day; 8 weeks	Antidepressant, Anti-inflammation, Antioxidant	↓ Tau phosphorylation	Choudhary et al. (2017)
Plant	MCT oil extract	*Zanthoxylum armatum* DC.	82	2.8 g/day; 1, 3, 5 h, 56 days	Anti-inflammation, Antioxidant	↓ Oxidative stress	Kennedy et al. (2019)
Plant	Capsule of ES, DR extract	*Eleutherococcus senticosus, Drynaria fortunei*	31	223 mg/day; 12 weeks	Antioxidant Anti-stress	Minimizing neuronal damage impreoved cognitive function	Tohda et al. (2020)
Plant	GRAPE dissolved in hot water	GRAPE formula	344	220 g/day; 24 months	Anti-inflammation, Antioxidant	↓ Oxidative stress ↑ ACh levels	Shi et al. (2017)
Plant	Huannao Yicong granules	HYE formula	52	10 g/day; 6 months	Anti-oxidant	↓AchE, Aβ_42_	Yang et al. (2019)
Plant	Jiannao Yizhi granules	JYF ormula	51	10 g/day; 6 months	Anti-inflammation, Antioxidant)	↑ACh ↓Aβ_42,_ Tau	Wang et al. (2020)
Plant	Kihito extract	Kihito	11	7.5 g/day; 16 weeks	Anti-oxidant	Promotes neuronal survival	Watari et al. (2019)
Plant	Nao-Xue-Shu oral liquid	Nao-Xue-Shu	158	30 mL/day; 2 weeks	Anti-inflammation	↓ IL-6, TNF-*α*	Jiang et al. (2016)
Plant	Yokukansan granules	Yokukansan	145	7.5 g/day; 4, 12 weeks	Antidepressant, Anticonvulsan, Antipsychotic	↓ TNF-α, IL-1β	Furukawa et al. (2017)

LDL, low density lipoprotein; EGb761, standardized extract of *Ginkgo biloba*; ChEls, cholinesterase inhibitors; MCT, medium-chain triglyceride; CBF, cerebral blood flow; PM-EE, PhytoMeal-ethanol extract; ES, *eleutherococcus senticosus*; DR, *drynaria fortune*; GRAPE, Ren shen (*Panax ginseng*, 10 g/d); Di huang (*Rehmannia glutinosa*, 30 g/d); Cang pu (*Acorus tatarinowii*, 10 g/d); Yuan zhi (*Polygala tenuifolia*, 10 g/d); Yin yanghuo (*Epimedium brevicornu*, 10 g/d); AchE, acetylocholine esterase; Ach, acetylcholine; SBI, secondary brain insult; HCH, hypertensive cerebral hemorrhage.

#### 2.21.1 Therapeutic investigations

Several clinical trials have highlighted the promising potential of natural compounds in AD therapy. For instance, diosgenin-rich yam extract, derived from *Dioscorea batatas*, demonstrated significant improvements in cognitive performance and semantic fluency in healthy adults. Efforts to optimize its hydrophobic nature have enhanced brain bioavailability, as evidenced by preclinical studies on ddY and 5×FAD mouse models (Tohda et al., 2017). Similarly, polyphenolic extract from Mentha spicata L. showed substantial cognitive benefits in patients with age-associated memory impairment (AAMI), with 900 mg/day supplementation significantly enhancing working memory, spatial accuracy, mood, and alertness (Herrlinger et al., 2018). The extract of *Salicornia europaea* L., known for its neuroprotective properties, was found to improve spoken language comprehension and daily cognitive function in MCI patients over a 6–12-week trial, with no reported adverse effects (Lee et al., 2020). Wild blueberry extract, evaluated by Whyte et al., was shown to improve episodic memory and reduce systolic blood pressure, demonstrating its dual cognitive and cardiovascular benefits (Whyte et al., 2018). In a placebo-controlled trial, *Withania somnifera* (Ashwagandha), administered at 600 mg/day for 8 weeks, significantly improved memory, executive function, and attention in patients with mild memory impairment, underscoring its potential in early intervention (Choudhary et al., 2017). Similarly, *Zanthoxylum armatum* DC. oil extract enhanced cognitive function and modulated cerebral blood flow, leading to increased task efficiency and reduced hemodynamic stress (Kennedy et al., 2019). The combined extract of *Eleutherococcus senticosus* and *Drynaria fortunei* demonstrated benefits in memory recall, language fluency, and stress resilience, indicating the value of combined therapies in enhancing cognitive health (Tohda et al., 2020). Furthermore, the GRAPE formula (*Panax ginseng*, *Rehmannia glutinosa*, *Acorus tatarinowii*, *Yuan Zhi*, and *Epimedium brevicornu*) showed sustained cognitive improvements in dementia patients, surpassing conventional therapies over a 24-month period (Shi et al., 2017). Both Huannao Yicong formula (HYF) and Jiannao Yizhi formula (JYF) demonstrated significant efficacy in reducing AChE and Aβ42 levels while improving cognitive function (Yang et al., 2019). HYF showed superior efficacy compared to donepezil, while JYF reduced tau protein levels, providing comparable benefits to standard ChEIs (Wang et al., 2020). The Kihito formula, when combined with ChEIs therapy, significantly improved cognitive scores in Mini-Mental-State Examination Japanese (MMSE-J) evaluations, surpassing monotherapy outcomes (Watari et al., 2019). Nao-Xue-Shu formula, targeting hypertensive cerebral hemorrhage patients, effectively reduced secondary brain injury symptoms and lowered inflammatory markers like IL-6 and TNF-α (Jiang et al., 2016). Lastly, Yokukansan alleviated behavioral and psychological symptoms of dementia, including agitation, aggression, and hallucinations, particularly in patients with severe cognitive impairment (MMSE <20) (Furukawa et al., 2017). Collectively, these clinical studies underscore the diverse therapeutic potential of natural compounds in addressing AD pathology and related symptoms. By modulating key pathways such as neuroinflammation, oxidative stress, and neurotransmitter regulation, these interventions offer promising complementary or alternative therapeutic strategies for AD management. Future large-scale, multicenter trials with standardized protocols are essential to validate these findings and facilitate the integration of natural products into mainstream AD treatment frameworks.

## 3 The role of herbal medicine in combating Alzheimer's disease

AD is a complex neurodegenerative disorder with multifactorial pathological mechanisms, including amyloid-β (Aβ) aggregation, tau hyperphosphorylation, neuroinflammation, oxidative stress, mitochondrial dysfunction, and synaptic loss. Conventional pharmacological treatments, such as ChEIs and NMDA receptor antagonists, primarily offer symptomatic relief without modifying the disease course (Breijyeh and Karaman, 2020; Peng et al., 2023). This limitation has fueled the search for alternative therapeutic strategies, with herbal medicine emerging as a promising approach due to its multi-targeted effects, low toxicity, bioavailability, and neuroprotective potential. Furthermore, AMP-activated protein kinase (AMPK) activation, a critical pathway regulated by several herbal compounds, has shown promise in preventing Aβ toxicity and preserving neuronal homeostasis. Herbal extracts such as *Withania somnifera*, *Panax ginseng*, *Ginkgo biloba*, and *Bacopa monnieri* exhibit multi-targeted benefits, positioning them as potential therapeutic agents in AD management (Martin et al. 2013; Park et al. 2016; Zhao et al. 2024).

*In vitro* models provide an essential platform for screening herbal compounds against AD-related pathology. Several studies have demonstrated that natural extracts effectively target Aβ fibrillation, oxidative stress, and neuroinflammation. These studies confirm that herbal compounds exert protective effects at the cellular level, offering potential as disease-modifying treatments. *In vivo* studies have validated the efficacy of herbal medicines in reducing Aβ accumulation, tau hyperphosphorylation, neuroinflammation, and cognitive impairment across various AD models, including APP transgenic mice, BALB/c mice, 5×FAD mice, SAMP8 mice, ICR mice, Kunming mice, and C57BL/6 mice. Herbal formulations such as *Ershiwuwei Shanhu* Pills (ESP), *Lycium barbarum* (LB), and *Bacopa floribunda* (BF) have shown efficacy in mitigating Aβ-induced neurotoxicity, oxidative stress, and mitochondrial dysfunction in BALB/c mice (Hu et al. 2018; Luo et al. 2022; Oyeleke and Owoyele 2022). Non-human primate (NHP) models, such as rhesus monkeys, provide a closer representation of human AD pathology, particularly in studying neuroinflammation and immune responses. Imaging modalities like positron emission tomography (PET) and two-photon microscopy have enhanced the translational relevance of herbal interventions (Li et al., 2019; Bjorkli et al., 2020; Fulop et al., 2021). Given the absence of a definitive cure for AD, multi-targeted therapeutic strategies have become increasingly necessary. While TCM holds considerable promise, its clinical application is often limited by poor bioavailability and restricted penetration of the BBB. Nanotechnology-based delivery systems, collectively referred to as nano-TCM, enhance the targeted transport of bioactive compounds, thereby improving therapeutic efficacy (Song et al., 2025). In one investigation, researchers identified 146 bioactive compounds from *Callicarpa kwangtungensis* (CK) that exert neuroprotective effects in AD models. These compounds were shown to enhance cognitive performance, reduce amyloid beta (Aβ) and tau pathology, and attenuate neuroinflammation. Mechanistically, CK modulates the tricarboxylic acid (TCA) cycle through the PI3K-AKT pathway and inflammation-related MAPK/NF-κB signaling, suggesting its therapeutic potential (Liu et al., 2025). Tau hyperphosphorylation, mediated by glycogen synthase kinase-3β (GSK-3β), is a key driver of progressive cognitive decline in AD. Natural compounds such as resveratrol and berberine have demonstrated the ability to modulate GSK-3β activity, thereby reducing tau pathology (Wang M. et al., 2025). Curcumin, widely known for its anticancer activity, also exerts neuroprotective effects by modulating microRNA expression and (Rahman et al., 2024) inhibiting key pathogenic pathways in AD (Wang X. et al., 2025). Other promising bioactive agents, including salviolone and vestitol, have been shown to influence calcium signaling and neuroactive ligand–receptor interactions, contributing to cognitive improvement and reduced Aβ deposition in experimental models (Liang et al., 2025). Marine-derived natural products have also emerged as viable therapeutic candidates in AD, with GV-971 demonstrating both clinical relevance and low toxicity. Additional compounds from marine sources have exhibited neuroprotective properties, supporting neuronal survival and cognitive function (Jia et al., 2025). A wide range of TCM-derived compounds have been studied for their neuroprotective effects in AD (Zhang et al., 2015). Notable examples include *Polygala tenuifolia*, *G. biloba*, and baicalin, which exhibit anti-Aβ aggregation and anti-inflammatory properties (Yancheva et al., 2009; Müller et al., 2019; Thancharoen et al., 2019). In addition, essential oils from plants such as lavender, thyme, and sage—including Spanish sage—have been shown to inhibit AChE and enhance cognitive function (Perry et al., 2001). The TCM component *Atractylodes lancea* DC has demonstrated potential as an adjuvant therapy when used alongside conventional pharmaceutical agents. Studies suggest that it may reduce mortality, mitigate oxidative stress and neuroinflammation, preserve BBB integrity, and improve cognitive function. Its anticancer activity further supports its potential for integrative applications in both oncological and neurodegenerative diseases (Plirat et al., 2023; Ahn et al., 2025). Additionally, various herbal products from Southeast Asia—such as rosemary—have shown neuroprotective properties by preventing encephalitis and suppressing Aβ accumulation. Phytonutrients including flavonoids and resveratrol, along with herbal extracts like ginseng, *G. biloba*, *B. monnieri*, and garlic-derived allicin, contribute to enhanced neuroprotection and reduced Aβ burden, thereby supporting cognitive health (Baranowska-Wójcik et al., 2025).

### 3.1 Addressing unresolved challenges and bridging research gaps in human studies on herbal treatments for Alzheimer’s disease

Clinical trials highlight the therapeutic potential of natural products in AD patients, demonstrating improvements in memory, cognitive function, and neuropsychiatric symptoms. Diosgenin-rich yam extract (Dioscorea batatas) improves cognitive performance and semantic fluency in healthy adults (Tohda et al., 2017). *Mentha spicata* L. (Spearmint extract) enhances working memory, spatial accuracy, and mood in patients with AAMI (Herrlinger et al., 2018). *Withania somnifera* (Ashwagandha) significantly improves executive function, attention, and memory in individuals with MCI (Choudhary et al., 2017). GRAPE formula (*P. ginseng, Rehmannia glutinosa, Acorus tatarinowii, Yuan Zhi, Epimedium brevicornu*) demonstrates sustained cognitive improvements over 24 months (Shi et al. 2017). Huannao Yicong formula (HYF) and Jiannao Yizhi formula (JYF) reduce Aβ42 and tau protein levels, delivering comparable benefits to standard ChEIs (Yang et al., 2019; Wang et al., 2020). Yokukansan alleviates agitation, aggression, and hallucinations in severe AD patients (MMSE <20) (Furukawa et al., 2017). These clinical findings suggest that herbal formulations can complement or enhance conventional AD therapies, with lower side effects and improved safety profiles. However, larger randomized controlled trials (RCTs) with standardized methodologies are necessary for regulatory approval and clinical integration. The development of effective therapeutic strategies for AD necessitates addressing key research gaps and unresolved challenges in human studies involving herbal remedies. Despite promising results from preclinical investigations, clinical studies often face issues related to poor bioavailability, inconsistent dosing, and methodological variability. To improve efficacy and reproducibility, future research should focus on the standardization of herbal compounds, optimization of clinical trial design, and adoption of integrative approaches that combine herbal therapies with conventional pharmacological treatments. Moreover, advancing personalized medicine in AD will require the incorporation of pharmacogenomic profiling to assess individual responses to natural compounds. These efforts will facilitate the translation of herbal-based interventions into clinically effective and scientifically validated treatments for AD.

### 3.2 Trends in FDA-approved drugs for Alzheimer’s disease

The development of pharmacological treatments for AD has been a significant focus of medical research, yet current FDA-approved drugs remain limited in their ability to modify the disease’s progression. Existing treatments primarily fall into two categories, ChEIs and NMDA receptor antagonists. ChEIs function by preventing ACh degradation within synaptic gaps, thereby enhancing neuronal communication and cognitive function. NMDA receptor antagonists, in contrast, work by reducing excitotoxicity, which can contribute to neuronal apoptosis and cognitive decline (Breijyeh and Karaman, 2020; Peng et al., 2023). Despite their benefits, these drugs provide only symptomatic relief and do not directly address the underlying mechanisms driving AD pathology, highlighting an urgent need for novel therapeutic strategies (Passeri et al., 2022). One major avenue of exploration has been monoclonal antibody therapies targeting amyloid beta (Aβ), such as donanemab and lecanemab. While these drugs show potential in reducing amyloid plaque burden, they come with risks such as amyloid-related imaging abnormalities with edema (ARIA-E), which can pose significant challenges to their widespread clinical use (Kurkinen, 2023; van Dyck et al., 2023; Høilund-Carlsen et al., 2024). Other FDA-approved drugs, including donepezil, galantamine, rivastigmine, and memantine, have demonstrated cognitive benefits but are frequently associated with gastrointestinal disturbances, dizziness, agitation, and, in some cases, severe hepatotoxicity, as observed with tacrine (Marucci et al., 2021; Peng et al., 2023; Varadharajan et al., 2023) (Table 16).

**TABLE 16:** FDA approved drugs for Alzheimer’s disease treatment.

Drug	Mechanism of action	Efficacy	Side effects	References
Donanemab	Anti-Aβ monoclonal antibody	Reduces amyloid plaques	ARIA-E	Kurkinen (2023), Høilund-Carlsen et al. (2024)
Donepezil	Acetylcholinesterase (AChE) inhibitor	Enhances cognition	Nausea, diarrhea	Adlimoghaddam et al. (2018); Marucci et al. (2021)
Galantamine	AChE inhibitor	Improves memory function	Nausea, diarrhea	Marucci et al. (2021)
Lecanemab	Anti-Aβ monoclonal antibody	Reduces amyloid plaques	ARIA-E	Söderberg et al. (2023); van Dyck et al. (2023)
Memantine	NMDA receptor antagonist	Reduces neurotoxicity	Dizziness, agitation, falls	Bago Rožanković et al. (2021); Varadharajan et al. (2023)
Rivastigmine	AChE and butyrylcholinesterase (BuChE) inhibitor	Improves cognitive function	Nausea, confusion, blurred vision	Marucci et al. (2021); Varadharajan et al. (2023)
Tacrine	AChE inhibitor (withdrawn)	Formerly used for AD	Severe hepatotoxicity	Marucci et al. (2021); Peng et al. (2023)

AChE, acetylcholinesterase; BuChE, butyrylcholinesterase; ARIA-E, amyloid-related imaging abnormalities with edema; NMDA, N-methyl-D-aspartate.

#### 3.2.1 Limitations of current AD treatments and the need for new approaches

Despite continuous advancements in AD drug development, currently available treatments do not effectively halt or reverse disease progression. The high failure rates of experimental drugs in late-stage clinical trials highlight a disconnect between preclinical findings and human studies, largely due to inadequate disease models that fail to capture the full complexity of human AD pathology (Blaikie et al., 2022). This emphasizes the pressing need for more translational research approaches that align with real-world disease progression. A growing body of evidence suggests that natural products may offer multi-targeted therapeutic potential, as demonstrated by numerous *in vitro* and *in vivo* studies showing their efficacy in reducing oxidative stress, neuroinflammation, and Aβ accumulation (Andrade et al., 2019). Despite promising preclinical results, no natural compound has yet received FDA approval for AD treatment, largely due to limitations in clinical study designs, inconsistent dosing regimens, and the absence of standardized protocols (Mohamed Yusof and Mohd Fauzi 2024).

## 4 Discussion

As the global population continues to age, the urgency to develop effective therapeutic strategies against AD has never been more critical. AD is a complex, progressive neurodegenerative disorder characterized by cognitive decline, amyloid-beta (Aβ) plaques, and tau protein neurofibrillary tangles. Despite decades of research, no cure exists, and current treatments focus primarily on symptom management rather than altering the disease trajectory (Ben-Shlomo et al., 2024; Sun and Zhang, 2024).

### 4.1 Re-evaluating therapeutic approaches in AD

For years, the amyloid cascade hypothesis has been the dominant framework guiding AD research. However, the repeated failure of Aβ-targeted therapies in clinical trials highlights the limitations of focusing on a single pathological mechanism. The growing understanding of AD as a multifactorial disease necessitates an approach that simultaneously addresses tau pathology, neuroinflammation, oxidative stress, mitochondrial dysfunction, and synaptic loss (Chu et al., 2024). Emerging research suggests that combination therapies, multi-targeted drugs, and personalized interventions hold greater potential in modifying AD progression compared to single-target treatments.

### 4.2 The role of neuroinflammation in AD pathogenesis

Recent evidence underscores neuroinflammation as a central player in AD progression. Chronic inflammation, primarily driven by activated microglia and astrocytes, exacerbates neuronal damage, accelerates synaptic dysfunction, and contributes to cognitive decline. Targeting neuroinflammatory pathways is now recognized as a promising therapeutic strategy, with potential benefits including restoration of neuronal function, promotion of neuroregeneration, and mitigation of Aβ and tau toxicity (Guo et al., 2020). However, achieving successful neuroinflammatory modulation remains challenging, requiring a delicate balance between suppressing harmful inflammation while preserving protective immune responses.

### 4.3 Challenges of current FDA approved treatments

Despite continuous research efforts, the efficacy of current FDA-approved drugs remains limited. ChEIs (e.g., donepezil, galantamine, rivastigmine) and NMDA receptor antagonists (e.g., memantine) primarily provide symptomatic relief, but they do not halt disease progression. Moreover, these drugs are often associated with side effects such as gastrointestinal distress, dizziness, and nausea, further limiting their long-term clinical utility (Alzheimer’sAssociation, 2022). Recent advances in monoclonal antibody therapies targeting Aβ plaques (e.g., donanemab, lecanemab) have shown potential, but they also pose significant risks, such as amyloid-related imaging abnormalities with edema (ARIA-E), highlighting the need for safer and more effective alternatives.

### 4.4 Socioeconomic burden and the need for cost-effective therapies

Beyond its medical impact, AD imposes a substantial socioeconomic burden, affecting patients, caregivers, and healthcare systems. In the United States alone, unpaid caregiving costs were estimated at $271 billion in 2021, underscoring the urgency of developing cost-effective treatments that not only alleviate symptoms but also slow disease progression (Alzheimer’sAssociation, 2022). Given the rising prevalence of AD, particularly in aging populations, innovative therapeutic approaches that integrate natural compounds, computational drug discovery, and precision medicine offer a sustainable and practical path forward.

### 4.5 The promise of natural compound in AD therapy

Natural compounds have garnered significant attention for their multi-targeting properties, low toxicity, and long-standing use in traditional medicine. Preclinical and clinical studies have demonstrated that bioactive compounds such as polyphenols, alkaloids, terpenoids, saponins, and flavonoids exert beneficial effects on AD-related pathways by reducing oxidative stress, suppressing neuroinflammation, preventing Aβ aggregation, and modulating tau hyperphosphorylation (Liu et al., 2024). Notably, certain plant-derived metabolites activate neuroprotective signaling pathways, such as the MAPK/NF-κB system, (Md et al., 2021), and interact with estrogen receptor beta (ERβ), a known regulator of synaptic plasticity and neuroprotection (Cardona-Gómez et al., 2006). The ability of these compounds to modulate multiple aspects of AD pathology makes them valuable candidates for future therapeutic interventions.

### 4.6 Overcoming challenges in translating natural compounds into clinical applications

Despite promising findings, translating the therapeutic potential of natural compounds into clinical practice presents several key challenges. Limited bioavailability and BBB penetration, reducing their central nervous system efficacy. Lack of standardization in formulation and dosing, leading to inconsistent study outcomes. Potential off-target effects and drug interactions, requiring more extensive toxicological profiling. Absence of large-scale clinical trials, delaying regulatory approvals and mainstream adoption (McGleenon et al., 1999). To address these barriers, advanced drug delivery systems (e.g., nanoformulations, liposomal carriers, prodrugs) are being explored to enhance bioavailability and targeted drug delivery to the brain. Additionally, network pharmacology and artificial intelligence (AI)-driven drug discovery are helping to optimize lead compounds and improve the efficiency of drug development pipelines. In addition, natural products are well recognized for their multi-target effects, a characteristic common to most herbal medicines. However, elucidating their precise molecular mechanisms of action remains a substantial challenge (Yang et al., 2023). This underscores the need for advanced research methodologies capable of addressing the complexity of translating preclinical findings into clinically validated treatments for AD using natural compounds (Ma et al., 2024b).

### 4.7 Integrating precision medicine and natural therapies

The rise of precision medicine, biomarker-driven diagnostics, and computational modeling is reshaping how AD is diagnosed and managed. Advances in fluid-based biomarkers (Aβ, tau, neurofilament light chain), functional neuroimaging (PET, MRI), and genetic profiling (APOE status, transcriptomics) are enabling earlier diagnosis and more personalized treatment strategies. The integration of natural compound research with these advanced diagnostic technologies represents a transformative shift, moving toward personalized and preventive therapeutic approaches rather than reactive symptomatic management.

### 4.8 Translational challenges and future directions

Despite promising preclinical findings, translating the therapeutic potential of natural compounds into clinically effective treatments for AD remains a significant challenge. Natural products frequently encounter substantial obstacles, including low bioavailability, limited penetration across the BBB, and inconsistent clinical trial outcomes, as exemplified by studies involving curcumin. These limitations have contributed to the lack of conclusive clinical efficacy observed to date (Kurkinen, 2023). Additionally, clinical trials involving natural products often face methodological issues such as inconsistent formulations, inter-individual variability among participants, and a need for large-scale studies to robustly assess therapeutic outcomes. Beyond pharmacokinetic and methodological constraints, regulatory barriers further impede the clinical translation of natural compounds for AD therapy. Challenges include batch-to-batch variability, absence of standardized extraction protocols, and inconsistent concentrations of bioactive constituents, all of which complicate compliance with Good Manufacturing Practice (GMP) standards and hinder reproducibility in clinical trials. Furthermore, most herbal products are classified as dietary supplements rather than pharmaceutical agents, limiting their regulatory oversight and permissible therapeutic claims. Regulatory authorities such as the U.S. FDA require stringent evidence of efficacy, safety, and quality control for multi-component natural extracts—criteria that are often difficult to fulfill. To overcome these obstacles, the establishment of harmonized international regulatory frameworks, validated analytical methodologies, and rigorously designed large-scale trials is essential. Addressing these issues is critical for the integration of natural compounds into precision medicine approaches for AD and for advancing their recognition as credible therapeutic agents within evidence-based clinical practice.

### 4.9 Addressing publication bias and negative outcomes

While the majority of studies cited in this review report positive findings regarding the effects of natural compounds in AD models, the potential influence of publication bias must be acknowledged. Specifically, studies with favorable outcomes are more likely to be published than those reporting negative or null results, leading to a skewed perception of therapeutic efficacy. Although this review has emphasized the promising potential of natural compounds, it is important to recognize that inconclusive or unfavorable data—particularly from preclinical studies involving transgenic mouse and zebrafish models—also exist. In preparing this review, we selected the most robust and relevant preclinical data to illustrate the therapeutic promise of natural products. However, we acknowledge that studies with conflicting or non-replicable findings are equally important and should be included in comprehensive future analyses. A balanced perspective is essential for identifying compounds whose benefits may be overestimated or insufficiently validated. Incorporating studies with negative or null results will help mitigate publication bias and contribute to a more accurate, evidence-based understanding of the role of natural products in AD. To achieve this, we encourage the scientific community to prioritize transparency in data reporting and to support the publication of all findings—regardless of outcome—in order to foster rigorous and reproducible research in the field of natural compound-based AD therapeutics.

## 5 Conclusion

AD remains one of the most formidable global health challenges, particularly among aging populations. Decades of research have expanded our understanding of its complex pathogenesis, yet current treatments offer only temporary symptomatic relief without significantly altering disease progression. The repeated failures of Aβ-targeted therapies highlight the need for a paradigm shift toward multi-targeted treatment approaches that address tau pathology, neuroinflammation, oxidative stress, mitochondrial dysfunction, and synaptic degeneration. Natural compounds offer a promising alternative, given their ability to modulate multiple pathways simultaneously. Our analysis presents a structured framework that elucidates the complementary and distinct mechanisms through which natural compounds contribute to AD treatment. By situating these phytochemicals within key pathological pathways, this integrative approach enhances our understanding of how multi-target agents can augment existing therapies and serve as a foundation for innovative, holistic strategies in AD management. Extensive research involving transgenic mouse models (APP, 5×FAD, SAMP8), *Drosophila melanogaster*, and human clinical trials has demonstrated their potential in reducing Aβ burden, improving cognitive function, and restoring neuronal health. However, bioavailability issues, lack of clinical standardization, and insufficient large-scale trials remain key obstacles that must be addressed before natural compounds can be integrated into mainstream AD therapy. Moving forward, combining natural product research with computational drug discovery, precision medicine, and innovative drug formulations will enhance therapeutic efficacy and accelerate clinical translation. Moreover, multi-targeted combination therapies that integrate synthetic and natural compounds could yield superior outcomes compared to single-target treatments. By integrating these diverse but complementary approaches, researchers and clinicians can move closer to effective disease-modifying therapies that not only alleviate symptoms but also slow or prevent AD progression, ultimately improving the quality of life for millions worldwide (Radhakrishnan et al., 2024). This review highlights the therapeutic potential of individual natural compounds, as well as emerging strategies in combinatorial and precision medicine, offering a comprehensive synthesis of current advancements in natural product-based AD therapy. Our aim is to enhance the translational relevance of natural product research in AD by developing innovative experimental platforms and bridging molecular mechanisms with contemporary clinical evidence.
